# Total flavonoids of hawthorn leaves protect spinal motor neurons *via* promotion of autophagy after spinal cord injury

**DOI:** 10.3389/fphar.2022.925568

**Published:** 2022-08-22

**Authors:** Qiong Zhang, Mingfu Liu, Haibin Nong, Yanan Zhang, Yiguang Bai, Pan Liu, Shaohui Zong, Gaofeng Zeng

**Affiliations:** ^1^ School of Public Health of Guangxi Medical University, Nanning, China; ^2^ Department of Spine Osteopathia, The First Affiliated Hospital of Guangxi Medical University, Nanning, China; ^3^ Collaborative Innovation Center of Guangxi Biological Medicine, Guangxi Medical University, Nanning, China; ^4^ Research Centre for Regenerative Medicine and Guangxi Key Laboratory of Regenerative Medicine, Guangxi Medical University, Nanning, China

**Keywords:** spinal cord injury, total flavonoids of hawthorn leaves, spinal cord motor neurons, autophagy, neuroprotection, axon regeneration

## Abstract

The death of spinal motor neurons (SMNs) after spinal cord injury (SCI) is a crucial cause, contributing to a permanent neurological deficit. Total flavonoids of hawthorn leaves (TFHL) have been confirmed to have potentially therapeutic for SCI. Nonetheless, the roles and mechanisms of TFHL in recovering neuromotor function and regenerating axons of SMNs have not been fully elucidated. In this study, TFHL was applied to treat rats with SCI and injured SMNs for 7 days. *In vivo* experiment, rats with SCI were evaluated by a BBB (Basso-Beattie-Bresnahan) score to assess their motor functional recovery. The morphology, microstructure, apoptosis, Nissl bodies, and autophagy of SMNs in spinal cord tissue were detected by Hematoxylin-eosin (HE) staining, transmission electron microscopy, TUNEL staining, Nissl staining, and immunohistochemistry respectively. *In vitro* experiment, the co-culture model of SMNs and astrocytes was constructed to simulate the internal environment around SMNs in the spinal cord tissue. The cell morphology, microstructure, axonal regeneration, and autophagy were observed via optical microscope, transmission electron microscopy, and immunofluorescence. The content of neurotrophic factors in the cell culture medium of the co-culture model was detected by ELISA. Moreover, the expression of axon-related and autophagy-related proteins in the spinal cord tissue and SMNs was measured by Western Blot. We demonstrated that TFHL improved the neuromotor function recovery in rats after SCI. We then found that TFHL significantly promoted injured spinal cord tissue repair, reduced apoptosis, and improved the functional status of neurons in spinal cord tissue *in vivo*. Meanwhile, the cell morphology, microstructure, and axonal regeneration of damaged SMNs also obviously were improved, and the secretion of neurotrophic factors was facilitated after treatment with TFHL *in vitro*. Further, we revealed that TFHL promoted autophagy and related protein expression *in vivo* and vitro. Taken together, our study suggested that TFHL might facilitate autophagy and have neuroprotective properties in SMNs to enhance the recovery of neuromotor function of rats with SCI.

## Introduction

Involvement of several cellular and molecular processes following spinal cord injury (SCI), closely related to the degree of neurological impairment, includes apoptosis, autophagy, inflammation, demyelination, axonal degeneration, and cell death (Kjell, 2016; Anjum et al., 2020). Among these, spinal motor neurons (SMNs) death after SCI results in demyelination of spared axons, even causing irreversible neuromotor function deprivation (Balaskas et al., 2019).

Given that SMNs are vital to functional recovery after SCI, our present study focused on the protection and restoration of damaged SMNs. In the spinal cord, the microenvironment in which SMNs survive maintains their normal growth and metabolic activities (Fan et al., 2018). After SCI, the spinal cord microenvironment continues to deteriorate, seriously interfering with the survival of SMNs, causing the secondary death of SMNs, and hindering the recovery of neuromotor function (Zhao et al., 2017). Therefore, it is crucial to improve the imbalance of the spinal cord microenvironment after SCI and maintain the stability of the external environment of SMNs to promote the repair of injured SMNs. Astrocytes are the most commonly distributed type of glial cells in the mammalian brain and spinal cord, which have many protrusions that stretch and fill between neurons to play a role in separating and supporting neurons (Giovannoni and Quintana, 2020). At present, astrocytes are considered to have an essential function in promoting the growth and development of neurons and maintaining the stability of the central nervous system microenvironment (Kisucka et al., 2021). Although past research has determined that hyperactivated astrocytes form glial scars after SCI, preventing nerve fibers from repairing and inhibiting neuronal axon growth (Okada et al., 2018). With the deepening of related research, many researchers have found that astrocytes can secrete neurotrophic factors and other neuronal support substances to endorse the repair of damaged neurons (Keller et al., 2020; Linnerbauer and Rothhammer, 2020). Furthermore, the degree of neuronal repair and its functional recovery is largely dependent on the growth of nascent axons. The increasing evidence supports the regeneration of axons is closely associated with autophagy (Ko et al., 2020; Crawley and Grill, 2021). Autophagy involves multiple molecular regulatory mechanisms, which are critical in maintaining the stability of the internal and external environment of cells and cell metabolism (Stavoe and Holzbaur, 2019). A large body of research indicates that proper up-regulation of autophagy can accelerate the replacement of damaged organelles in cells, enhance the functional repair of the injured spinal cord, and act as a positive protective factor (Ray, 2020; Liao et al., 2021). Thus, a new therapeutic strategy is urgently required to prevent the death of injured SMNs and ameliorate the spinal cord microenvironment following SCI.

Total flavonoids of hawthorn leaves (TFHL), an all-encompassing term for several types of flavonoids (Quercetin 3β-D-glucoside, rutin, catechin, quercetin, isorhamnetin, genistein, astragalin, luteolin, (-)-epicatechin, 3- Methoxy-5,7,3',4'-tetrahydroxy-flavonoids, etc.) derived from hawthorn leaves, have been reported to have anti-inflammatory effects, antioxidant activity, radical scavenging activity, and neuroprotective property (Wang D. J. et al., 2018). In our previous study, TFHL was confirmed to have neuroprotective effects on SCI by reducing cell apoptosis, decreasing neural tissue damage, and inhibiting inflammatory responses (Zhang et al., 2021). However, the effect of TFHL on the survival of injured SMNs and regeneration of axons after SCI remain unclear.

In this study, we investigated whether TFHL protects the injured SMNs and promotes the functional recovery of the spinal cord after SCI, and explored the possible mechanisms. We first revealed that TFHL improved the recovery of neuromotor function and the reconstruction of damaged spinal cord tissue following SCI *in vivo* experiments. We then demonstrated that TFHL significantly reduced the death of injured SMNs and assisted the regeneration of axons *in vitro* experiments. Meanwhile, we further evidenced that TFHL promoted the level of autophagy and ameliorated the microenvironment of injured spinal cord tissue, which might be tightly related to the survival of SMNs. Taken together, our study demonstrated that TFHL can protect SMNs via the promotion of autophagy after SCI.

## Materials and methods

### Experimental animals

45 Sprague-Dawley (SD) rats without specific pathogens, aged 4–6 weeks and weighing 200–250 g (half females, half males) and 80 newborn SD rats whose birth times cannot exceed 24 h were obtained from the Animal Experimental Center of Guangxi Medical University (license No. SCXK (Gui) 2014-0005). The adult rats were housed at 20–25°C with a relative humidity of 50–60% with a 12 h light/dark cycle and were supplied with standard food and water throughout the day. The Guangxi Medical University Ethics Committee approved all experimental procedures on animals (approval No. 201911015) in November 2019.

### Drugs and drug preparation

TFHL was obtained from a Chinese pharmaceutical company, Shanxi Kanglisheng Pharmaceutical Co., Ltd. (product batch No. 15118240). The information about TFHL was shown in Supplementary Table S1. In this study, poloxamer 188 (Well Chemical Co., Ltd., Nanjing, China) was used to increase the solubility of TFHL. First, weigh 100 g TFHL and screen them with an 800-mesh sieve to remove large particles of impurities. Second, TFHL and poloxamer 188 were mixed evenly in glassware according to the ratio of 1:4 and stirred vigorously in boiling water for 30 min. When TFHL and poloxamer 188 were completely melted, the substance was immediately cooled and quickly refrigerated at −20°C to solidify for 24 h, and then the solidified drug was dried, dispersed, and passed through a 60-mesh sieve to obtain the final TFHL.

### Spinal cord injury model establishment and drug administration

45 rats were randomly assigned into three groups: the Sham group, the SCI group, and the TFHL group. The SCI model was established using Allen’s method (Allen, 1911). In the procedure, rats were sedated intraperitoneally with 0.7 ml/100 g of 5% chloral hydrate (Maclin, Suffolk, UK), then fixed in the prone position. After that, the skins of rats were incised by 3 cm, and a separation of the subcutaneous fascia and paravertebral muscles was performed. Following the cutting of the ligaments of the interspinous process, the T9-11 spinous processes were completely exposed. By gently redirecting the ligamentum flavum, the T10 lamina was clamped with forceps to expose the dura mater. The Allen device (10 g Kirschner wire, Jiangzhou Medical Equipment Co., Ltd., Jiangsu, China) fell freely from a height of 3 cm, striking the dural sac and causing injury to the T10 segment of the spinal cord. Unlike the SCI group, the Sham group did not undergo any spinal cord damage to the T10 segment. After the operation, subcutaneous injections of 30,000 units of penicillin were given once every day to the rats, and bladder pressure was applied twice daily for 3 days for urination. When the hind limbs of the rats did not react to stimulation and the vital signs were stable, indicating that the SCI model had been successfully established. According to the dose of 50 mg/kg, the prepared TFHL was ultrasonically dissolved in 2 ml of normal saline to obtain the TFHL solution. The rats of the TFHL group were given 2 ml TFHL solution for intragastric administration, three times a day for 7 consecutive days. The Sham group and the SCI group were gavaged with the same amount of normal saline, and the administration frequency and time were the same as the TFHL group.

### Motor function test

To assess the extent of limb movement associated with the injured part of the spinal cord in rats at 1, 3, and 7 days post-injury, Basso-Beattie-Bresnahan (BBB) score was used (Basso et al., 1995; Basso et al., 1996). BBB scores are assigned from 0 (flat paralysis) to 21 (normal gait) according to the ankle, hip, knee, and trunk movements, as well as coordination. Three observers who were blinded to the treatments recorded the movements of the rats and the scores to calculate the average BBB score of each group.

### Hematoxylin-eosin staining

The spinal cord tissues of the T10 segments of rats from the three groups were excised and fixed in liquid paraffin (Sinopharm Group; 69019361, Shanghai, China) on day 7. After dewaxing in xylene (Sinopharm Group; 10023418) and hydrating with concentration gradient of ethanol, the tissue sections were stained with hematoxylin and eosin. Finally, Under an upright fluorescence microscope (Olympus BX53, Tokyo, Japan), the sections were observed.

### Transmission electron microscopy of spinal cord tissue

The spinal cord tissues of the T10 segments were fixed with 2.5% glutaraldehyde (Maclin; G849973, Suffolk, UK) at 4°C for 2 h. After washing three times with PBS, the spinal cord tissues were fixed again with 1% osmium tetroxide (Maclin; P816056) at 4°C for 2 h. Subsequently, the spinal tissues were dehydrated using ethanol gradients of 70%, 80%, and 90% (Sinopharm Group; 10009218) and 90 and 100% 1:1 ethanol and acetone (90% ethanol: 90% acetone) (Aladdin; S104174, Shanghai, China). The tissues were embedded with epoxy resin (Sigma-Aldrich, St. Louis, MO, United States; 430234) following infiltrating with propylene oxide, and then sliced into ultrathin sections. Saturated uranium acetate and lead citrate were applied to stain the sections for 30 and 8 min respectively (ALFA, Haverhill, MA, United States; A04A10701). And the sections were observed under a scanning transmission electron microscope (HITACHI H-7650, Tokyo, Japan).

### TUNEL staining

TUNEL apoptosis detection kits (Jiamay; TUN11684817, Beijing, China) were used to detect cell apoptosis in the spinal cord tissue. Following the instructions, Proteinase-k was applied to the tissues for 15 min at normal temperature, and then TUNEL reagent mixture solution was added for 1 h at 37°C. After the sections were air-dried, 50 µL of converter-POD was added to react with them in an incubator at 37°C for 30 min, followed by 50 µL of DAB substrate to react at 15°C for 10 min. In addition, the nuclei were stained with DAPI (Beyotime; C1002, Shanghai, China). Fluorescence microscopy (Olympus BX53, Tokyo, Japan) was used to observe the TUNEL-stained sections. Three researchers who were blinded to the treatments measured the percentage of TUNEL-positive cells in the spinal cord tissues of various groups.

### Nissl staining

After the tissue paraffin sections were dewaxed, hydrated, and washed with distilled water, they were stained with 0.25% toluidine blue (Sinopharm Group; 71041284) at 60°C for 3 h. The sections were then washed with 95% ethanol, dehydrated with absolute ethanol, made transparent with xylene (Sinopharm Group; 10023418), and eventually assembled with neutral gum (Sinopharm Group; 10004160). The sections were observed and images were captured under a vertical fluorescent microscope (Olympus BX53, Tokyo, Japan). The average optical density of Nissl bodies was analyzed by ImageJ 5.0 software (Rawak Software Inc., Stuttgart, Germany) by three researchers who were blinded to the treatments.

### Immunohistochemistry

After the tissue paraffin sections were dewaxed, hydrated, and incubated in a hydrogen peroxide solution for 1 h at room temperature, LC3-Ⅱ (1:400, Abcam, Cambridge, UK) antibodies were used to evaluate autophagy in the injured spinal cord. The sections were observed and images were captured under a vertical fluorescence microscope (Olympus BX53, Tokyo, Japan). The sum of the area and the optical density (IOD) of LC3-Ⅱ was measured by ImageJ 5.0 software (Rawak Software Inc., Stuttgart, Germany). The average optical density of LC3-Ⅱ is equal to the total IOD divided by the total area. The above analysis was completed by three researchers who were blinded to the treatments.

### Primary spinal motor neurons dissociation and culture

Primary SMNs were isolated from the spinal cord anterior horn tissue of 80 newborn SD suckling rats as described. Briefly, the spinal cord anterior horn tissue was dissected, mechanically dissociated, and then digested for 30 min with 2 mg/ml papain (Worthington Biochemical, Lakewood, United States) and a little DNase I (Thermo Fisher, Waltham, United States) at 37°C. The cell suspension was harvested and seeded on poly-D-lysine-coated cell slides in six-well culture plates (Corning, NY, United States) at a density of 7 cells/cm^2^ × 10^5^ cells/cm^2^ and cultured for 4 h in high glucose DMEM/F12 (Gibco, NY, United States) containing 10% FBS (Gibco, NY, United States) at 37°C with 5% CO_2_. To remove contaminating glial cells, the medium must be exchanged with specialized Neurobasal-A (Thermo Fisher, Waltham, United States) supplemented with 2% B27 (Gibco, NY, United States) and 2 mmol/L Glutamax (Thermo Fisher, Waltham, United States) after 4 h cell adhesion. The quality of spinal motor neurons was analyzed by immunostaining of β-Tubulin.

### Primary spinal astrocytes culture

Primary spinal astrocytes were obtained from Wuhan Procell Life Technology Co., Ltd. (product batch No. CP-R306). After stabilizing for 24 h in an incubator containing 5% CO_2_ at 37°C, the primary spinal astrocytes were seeded onto six-well culture plates in DMEM/F12 supplemented with 15% FBS at a density of 5 cells/cm^2^ × 10^5^ cells/cm^2^. The purity of astrocytes was identified by GFAP immunostaining.

### The injured spinal motor neurons model construction

Glutamate can cause neuronal excitability injury and simulate the mechanism of neuronal injury caused by spinal cord microenvironment imbalance after SCI (Meldrum, 2000). In this study, glutamate was used to construct a spinal motor neuron injury model. When the dendrites of SMNs were abundant and covered the whole-cell slide, Neurobasal-A medium containing 200 µM glutamate (Sinopharm Group, Shanghai, China) was used to interfere SMNs for 24 h. When it was found that the cell body of the neurons was retracted and the dendrites were broken but not floating in the medium, it indicates that the spinal motor neuron injury model has been successfully constructed. The medium containing glutamate was immediately removed and gently rinsed with PBS buffer twice, then replace with the fresh medium for cultivation.

### The co-culture model of spinal motor neurons and astrocytes and drug intervention

SMNs were randomly assigned to five groups: the Control group, the Hurt group, the Astrocyte group, the Direct drug group, and the Co-culture group. In the Control group, SMNs were cultured normally without any injury or intervention. There were only SMNs injured by glutamate in the Hurt group. There were the injured SMNs co-cultured with astrocytes but without TFHL intervention in the Astrocytes group. TFHL was used to intervene on the injured SMNs in the Direct drug group. Likewise, in the Co-culture group, TFHL was applied to intervene on the co-culture of injured SMNs and astrocytes.

After the injury model of SMNs was successfully constructed, take out the neuron-grown cell slides from the six-well culture plates, and buckle them on the 6-well culture plates with astrocytes. In the plates, the SMNs can be attached to astrocytes, and the original medium was replaced with a special Neurobasal-A medium. One day after co-culture, the prepared TFHL was ultrasonically dissolved in Neurobasal-A medium to obtain the high concentration TFHL solution, which was added to the cell culture wells to dilute the final solution to 125 µg/ml to interfere with damaged neurons for 7 days.

### Cell morphology observation

The morphological changes of SMNs and astrocytes in each group were observed under an optical microscope (Olympus CKX41, Tokyo, Japan) following 7 days of continuous treatment with TFHL. Observation of the morphology of SMNs required sterile micro-tweezers to remove the neuronal cell slides from the co-cultured culture plates and place them on new culture plates. After the observation was completed, put them back into the co-culture plates.

### Transmission electron microscopy of spinal motor neurons

To observe the changes in microstructure in SMNs after 7 days of continuous treatment with TFHL, SMNs were collected from cell slides in each group and centrifuged at 1,000 rpm for 2 min to gather mung bean-sized cells clusters. The preparation procedure of transmission electron microscopy of SMNs was consistent with the processing of spinal cord tissue.

### Immunofluorescence

Immunofluorescence staining for MAP2 and LC3-Ⅱ was performed. Initially, the cell slides of attached SMNs were rinsed in PBS for 5 min thrice and fixed in 4% paraformaldehyde for 15 min. Then, after rinsing thrice with PBS, the cell slides were blocked in 5% goat serum (Gibco, NY, United States) containing 0.3% Triton X-100 (Solarbio, Beijing, China) for 30 min at room temperature. Afterwards, the cell slides were incubated with the primary antibodies overnight at 4°C, followed by 2 h of incubation with the appropriate secondary antibodies at 37°C. Finally, the cell slides were counterstained with DAPI to label nuclei and coverslipped. Images were taken under fluorescence microscopy (BX51, Olympus, Tokyo, Japan). The following primary antibodies were used: rabbit microtubule Association Protein 2 (MAP2, 1:1,000, Abcam plc, Cambridge, UK), rabbit Light Chain 3-Ⅱ (LC3-Ⅱ, 1:500, Abcam plc, Cambridge, UK). The appropriate Alexa Flour® fluorescent secondary antibodies were obtained from Abcam plc in Cambridge, United Kingdom.

### Enzyme-linked adsorption assay

The content of neurotrophic factors in the cell culture medium of each group was detected by enzyme-linked adsorption assay (ELISA) after the continuous intervention of TFHL for 7 days. It was recommended that samples be diluted according to the instructions of the ELISA kit manufacturer (Boster, Wuhan, China). Using standard proteins, including brain-derived neurotrophic factor (BDNF), glial-derived neurotrophic factor (GDNF), nerve growth factor (NGF) and ciliary neurotrophic factor (CNTF), the samples were prepared in duplicates, and the immunoreaction method was followed according to the kit manufacturer’s protocol. The absorbance of the wells was measured with a spectrophotometer (Shanghai Spectrophotometer Co. Ltd., China) at 450 nm. After calculating the concentrations of BDNF, CNTF, NGF, and GDNF of each sample (pg/ml) divided by total protein concentrations (mg/ml), two ratios were calculated to identify how much BDNF, CNTF, NGF, and GDNF in each sample are present per milligram of total protein (pg/mg).

### Western blot assay

To detect the expression of related-proteins in spinal cord tissues and SMNs, approximately 1.5 cm injured spinal cord segments and SMNs were harvested respectively, and a pre-prepared lysate (PIRA lysate + PMSF protease inhibitor; Cell Signaling Technology, Danvers, MA, United States) was added, followed by centrifugation at 10,000 g for 15 min at 4°C to extract the supernatant. A BCA Protein Assay Kit (Beyotime, Shanghai, China) was used to determine the protein sample concentration before loading. After electrophoresis of proteins on polyacrylamide gel containing 10% sodium dodecyl sulfate, the proteins were transferred to nitrocellulose membranes, which were then blocked with Tris buffer containing tween 20 and nonfat dry milk for 1 h at room temperature. Afterward, the membrane was incubated with the primary antibody (rabbit GAPDH antibody, 2118S, 1:1,000; rabbit LC3, 12741S, 1:1,000, obtained from Cell Signaling Technology, Danvers, MA, United States rabbit SCG10 antibody, ab185956, 1:10000; rabbit P62 antibody, ab91526, 1:1,000; rabbit Beclin-1, ab210498, 1:1,000, obtained from Abcam, Cambridge, UK) overnight at 4°C, in which the rabbit GAPDH was served as an internal control. After incubating with the secondary antibody (goat anti-rabbit horseradish peroxidase-conjugated IgG, 1:5,000; Jackson Immunochemicals, West Grove, PA, United States) at room temperature for 1.5 h, the membranes were observed on a GE Amersham Imager 600 (General Electric, Boston, MA, United States). The gray value ratio of protein bands was quantified using ImageJ 5.0 software (Rawak Software Inc., Stuttgart, Germany).

### Statistical analysis

All experimental data were presented as the mean ± standard deviation $\left(\bar{x}\pm s\right)$ and analyzed three times at least with SPSS 24.0 statistical software (IBM, Armonk, NY, United States). One-way analysis and repeated measures analysis of variance was used to compare differences among the groups. For the further comparison of differences between the two groups, the post-test, the least-significant difference test, was used. It was considered that a value of *p* < 0.05 has statistically significant.

## Results

### Total flavonoids of hawthorn leaves promotes motor function recovery after spinal cord injury

**FIGURE 1:**
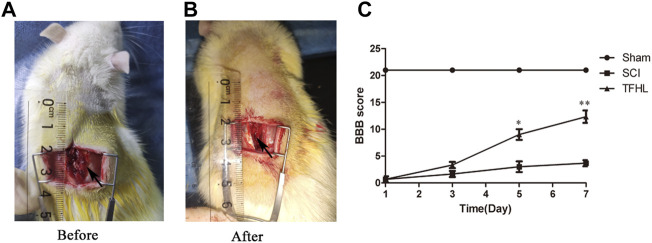
TFHL promotes motor function recovery after SCI. **(A,B)** The exposure of spinal cord tissue before and after injury in rats. **(C)** BBB scores of rats in each group at 1, 3, 5, and7 days post SCI. All rats in the Sham group exhibited BBB scores of 21 for the entire measurement period. The SCI group had the lowest BBB scores. The BBB scores in the TFHL group were considerably higher than those of the SCI group, in which the difference in BBB scores was the most obvious on day 7. All data are expressed as the mean ± SD. Differences among groups were determined with repeated measures ANOVA. *n* = 15 in each group, *p < 0.05, **p < 0.01.

The recovery of hindlimb motor function in rats was reflected by BBB scoring following treatment with TFHL. The BBB scores reached 21 in the Sham group, indicating good motor function. While the BBB scores decreased obviously in the SCI group, indicating impaired motor function (*p* < 0.05). The BBB scores were higher in the TFHL group than in the SCI group, and the scores increased in a dose-dependent manner with time, in which the effect of TFHL was most obvious on day 7 (*p* < 0.01, Figure 1).

### Total flavonoids of hawthorn leaves alleviates spinal cord tissue damage after spinal cord injury

**FIGURE 2:**
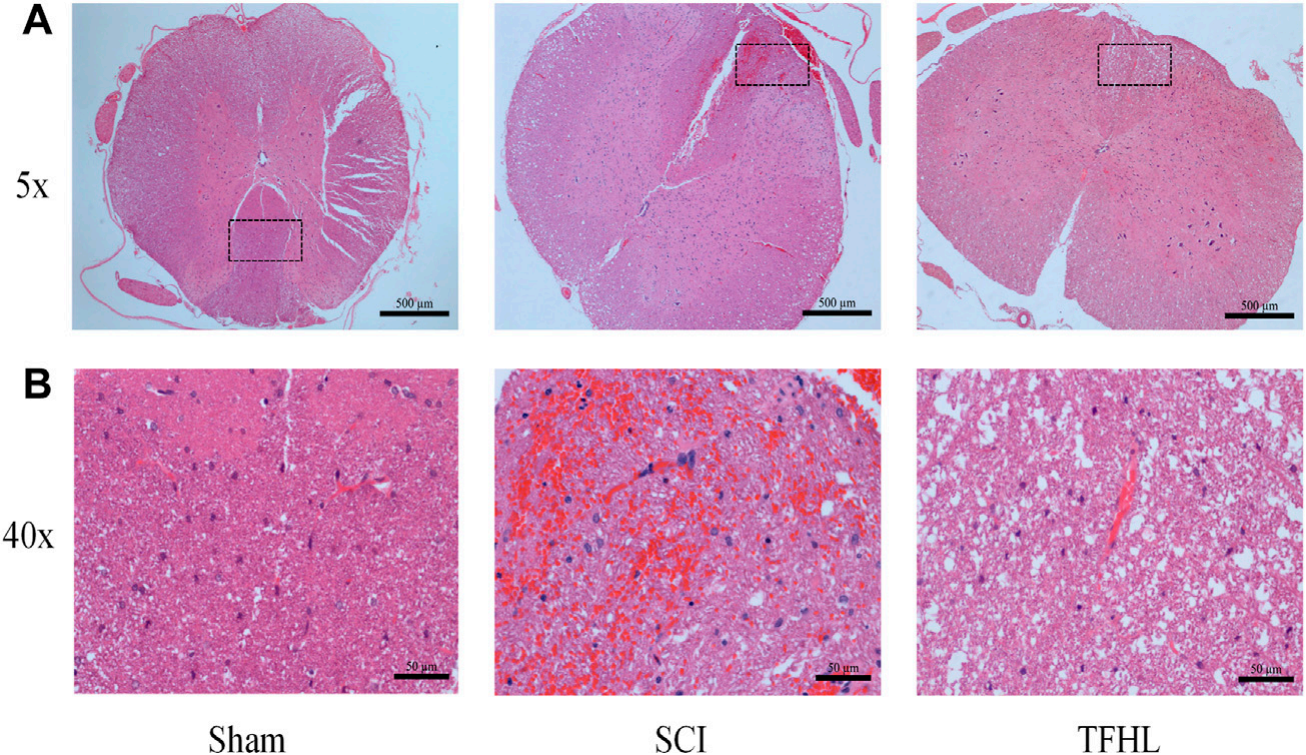
TFHL alleviates spinal cord tissue damage after SCI. Assessment of injury in spinal cord sections of each group by HE staining (*n* = 3 in each group, cross-section of the T10 segment of the spinal cord). **(A)** Under ×5 magnification, the overall structure of the spinal cord tissue of each group was observed, with scale bars = 500 μM. **(B)** Under ×40 magnification, the pathological changes of the injured spinal cord tissue in each group were seen, with scale bars = 50 μM. The black dashed box is the observation part under the ×40 magnification lens.

The changes in injured spinal cord tissue of rats were observed by HE staining following treatment with TFHL. The results of HE staining showed that at ×5magnification, the morphology of neurons in the gray matter of the spinal cord in the Sham group was normal, with large and full nuclei, which can clearly distinguish the morphological differences with astrocytes. The white matter structure of the spinal cord was complete, the nerve fibers were neatly arranged, and there was no bleeding point at ×40magnification. In the SCI group, the morphology of neurons in the gray matter of the spinal cord was disordered, the nucleus was retracted, the form of the neurons was difficult to distinguish, and there was a certain infiltration of inflammatory cells at ×5magnification. A large number of bleeding points were found in the white matter of the spinal cord, accompanied by obvious inflammatory cell infiltration at ×40magnification. Comparatively, the morphology of neurons in the spinal cord gray matter had improved in the TFHL group, and the difference between neurons and other cells was apparent, the bleeding point in the white matter of the spinal cord had been absorbed, and a cavity formed after the inflammation subsided at ×40magnification (Figure 2).

### Total flavonoids of hawthorn leaves facilitates microstructure and autophagosome change of injured spinal cord tissues

**FIGURE 3:**
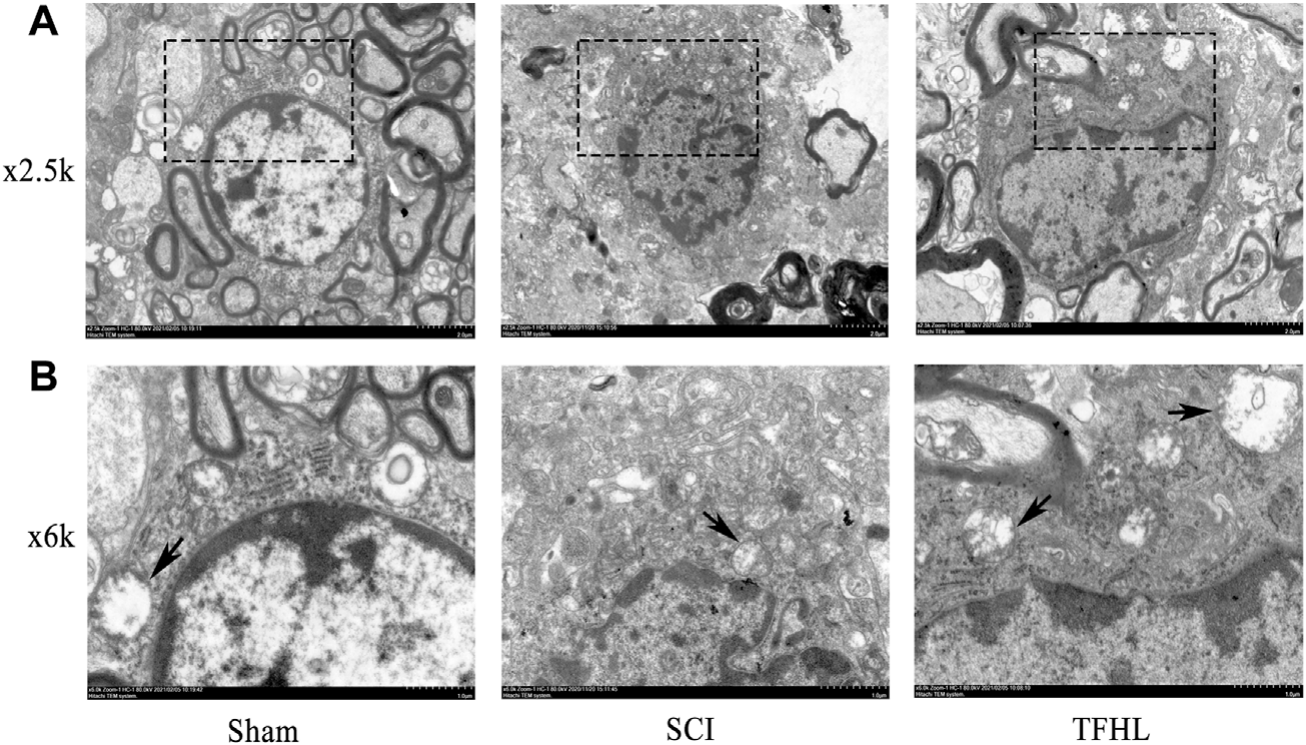
TFHL facilitates microstructure and autophagosome change of injured spinal cord tissues. Observation of microstructure and autophagosome change in spinal cord sections of each group by transmission electron microscopy (*n* = 3, cross-section of the T10 segment of spinal cord). **(A)** Under ×2.5 k electron microscope, the changes of neuronal nuclei and surrounding myelin sheaths in spinal cord tissue of each group were observed, with scale bars = 2 μM. **(B)** Under the ×6 k electron microscope, the autophagosomes in cells were found, with scale bars = 1 μM. The black dashed box is the observation site under ×6 k magnification, and the arrow points to the autophagosome.

The changes in the microstructure and autophagosomes of the injured spinal cord of rats were observed by transmission electron microscope following treatment with TFHL. Under ×2.5 k magnification, the nuclei of neurons in spinal cord tissue were large and round, and the surrounding myelin sheaths were arranged compactly, with complete morphology, and Schwann cells can be clearly seen in the myelin sheath in the Sham group. In the SCI group, the nuclei of the neurons were loose, and the surrounding myelin sheaths were broken. After treatment with TFHL, the nuclei of the neurons recovered to some extent, and the shape and arrangement of myelin sheaths tended to be normal. Under ×6 k magnification, it can be found that there are a few autophagosomes in neurons of the Sham group. When the spinal cord was injured, the autophagosomes in neurons increased. In contrast to the SCI group, the TFHL group had a higher percentage of autophagosomes (Figure 3).

### Total flavonoids of hawthorn leaves suppresses cell apoptosis in injured spinal cord tissues

**FIGURE 4:**
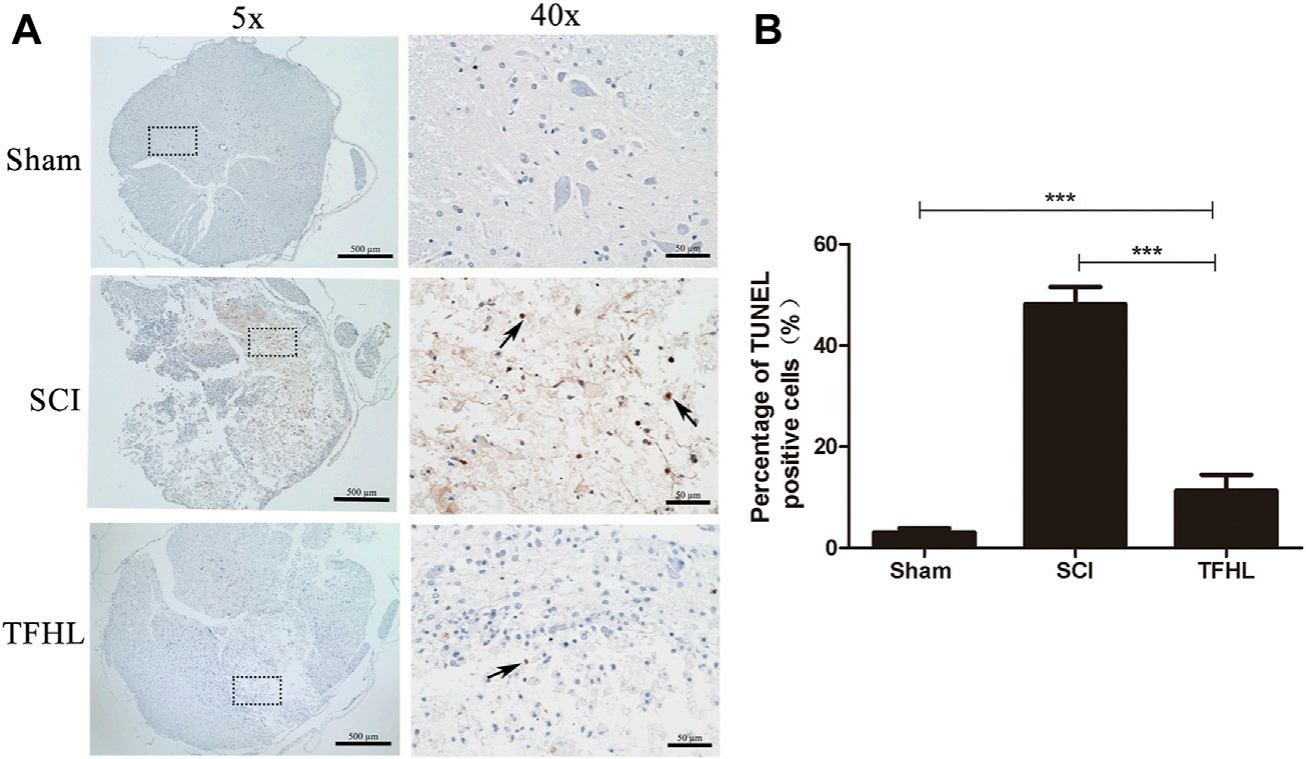
TFHL suppresses cell apoptosis in injured spinal cord tissues. **(A)** Observation of cell apoptosis in spinal cord sections of each group by TUNEL staining (*n* = 3, cross-section of the T10 segment of spinal cord, scale bars: 500 and 50 μM). Under the microscope, tawny represents apoptotic cells in the spinal cord tissue. The black dashed box in the ×5 magnification is the observation site under ×40 magnification, and the arrow points to TUNEL positive cells. **(B)** The percentage of TUNEL positive cells was counted in the spinal cord tissues of each group. Notice the decrease in the percentage of TUNEL positive cells the in TFHL group compared to the SCI group. All data are expressed as the mean ± SD. Differences among groups were determined with one-way ANOVA followed by a least-significant difference post hoc test. *p < 0.05, **p < 0.01, ***p < 0.001.

The cell apoptosis in the injured spinal cord tissues of rats was observed by TUNEL staining following treatment with TFHL. Under ×5 magnification, it can be found that the spinal cord tissue was deformed, gray and white matter were disordered, and a large number of TUNEL-positive cells appeared in the SCI group. While the spinal cord tissue morphology has been improved, and TUNEL-positive cells have been significantly reduced after treatment with TFHL (Figure 4A). A significantly higher percentage of TUNEL-positive cells was observed in the SCI group than that in the Sham group (*p* < 0.001). As compared to the SCI group, the THFL group had fewer TUNEL-positive cells in proportion to total cells. (*p* < 0.001, Figure 4B).

### Total flavonoids of hawthorn leaves improves the functional status of neurons in injured spinal cord tissues

**FIGURE 5:**
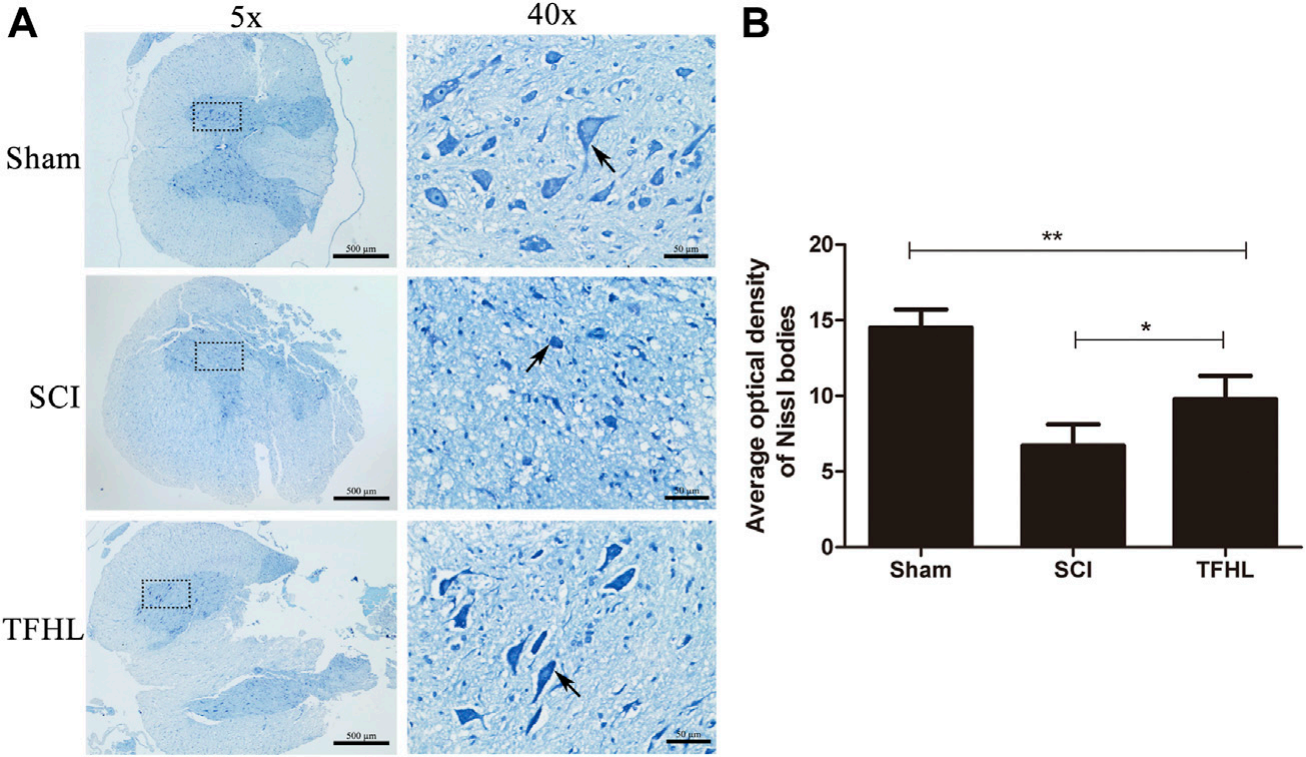
TFHL improves functional status of neurons in injured spinal cord tissues. **(A)** Observation of Nissl bodies in neurons of spinal cord tissues of each group by Nissl staining (*n* = 3, cross-section of the T10 segment of spinal cord, scale bars: 500 and 50 μM). Blue staining represents a Nissl body, as shown by the arrows. The darker the color of the Nissl bodies or the tabby shape, the better the neuron state. The black dashed box in the ×5 magnification is the observation site under ×40 magnification. **(B)** The average optical density of Nissl bodies in spinal cord tissues of each group. Notice the increase in average optical density of Nissl bodies in the TFHL group compared to the SCI group. All data are expressed as the mean ± SD. Differences among groups were determined with one-way ANOVA followed by a least-significant difference post hoc test. *p < 0.05, **p < 0.01, ***p < 0.001.

The functional status of neurons in injured spinal cord tissues of rats was observed by Nissl staining following treatment with TFHL. Under ×40 magnification, neurons in the spinal cord tissue of the Sham group were spindle-shaped, with complete structure, regular morphology, obvious nuclei, and abundant and stained Nissl bodies that arranged as a tabby in the cytoplasm. In the SCI group, the neurons were indistinguishable, the nuclei were not obvious, and the Nissl bodies in the cytoplasm were atrophied or smaller. As compared to the SCI group, the THFL group demonstrated significantly improved neuron morphology, as well as an increase in the number of Nissl bodies with a tabby shape (Figure 5A). In addition, the average optical density value of Nissl bodies was significantly lower in the SCI group than that of the Sham group (*p* < 0.01). While the average optical density value of Nissl bodies gradually increased in the TFHL group (*p* < 0.05, Figure 5B).

### Total flavonoids of hawthorn leaves promotes autophagy in injured spinal cord tissues

**FIGURE 6:**
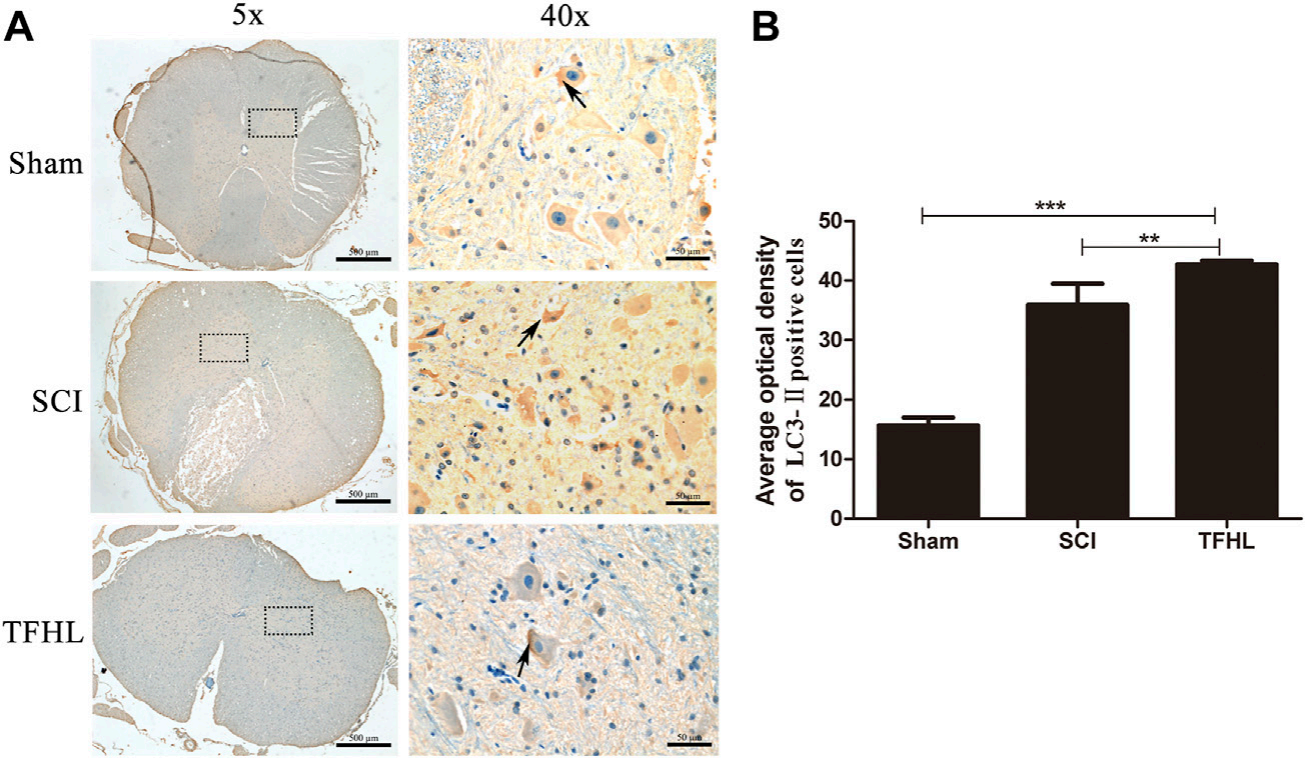
TFHL promotes autophagy in injured spinal cord tissues. **(A)** Observation of autophagy of spinal cord tissues of each group by Immunohistochemistry (*n* = 3, the cross-section of the T10 segment of the spinal cord, scale bars: 500 and 50 μM). Brown staining indicates the LC3-Ⅱ positive cells, as shown by the arrows. The black dashed box in the ×5 magnification is the observation site under ×40 magnification. **(B)** The average optical density of LC3-Ⅱ positive cells in spinal cord tissues of each group. All data are expressed as the mean ± SD. Differences among groups were determined with one-way ANOVA followed by a least-significant difference post hoc test. *p < 0.05, **p < 0.01, ***p < 0.001.

The expression of autophagy in injured spinal cord tissues of rats was detected by immunohistochemistry following treatment with TFHL. It can be found that the nuclei of the neurons in the spinal cord tissues were blue, and the cytoplasm was brown or even yellow-brown, which indicates that the LC3-Ⅱ autophagy-related protein was positive (Figure 6A). Analyze and compare the average optical density of LC3-Ⅱ in the spinal cord tissue of each group. The results show that the average optical density of LC3-Ⅱ positive cells in the spinal cord tissue of the SCI group increased compared with that of the Sham group (*p* < 0.001). The average optical density of LC3-Ⅱ positive cells in the spinal cord tissue of the THFL group was higher relative to the SCI group (*p* < 0.01, Figure 6B).

### Total flavonoids of hawthorn leaves influences protein expression linked to axons and autophagy in injured spinal cord tissues

**FIGURE 7:**
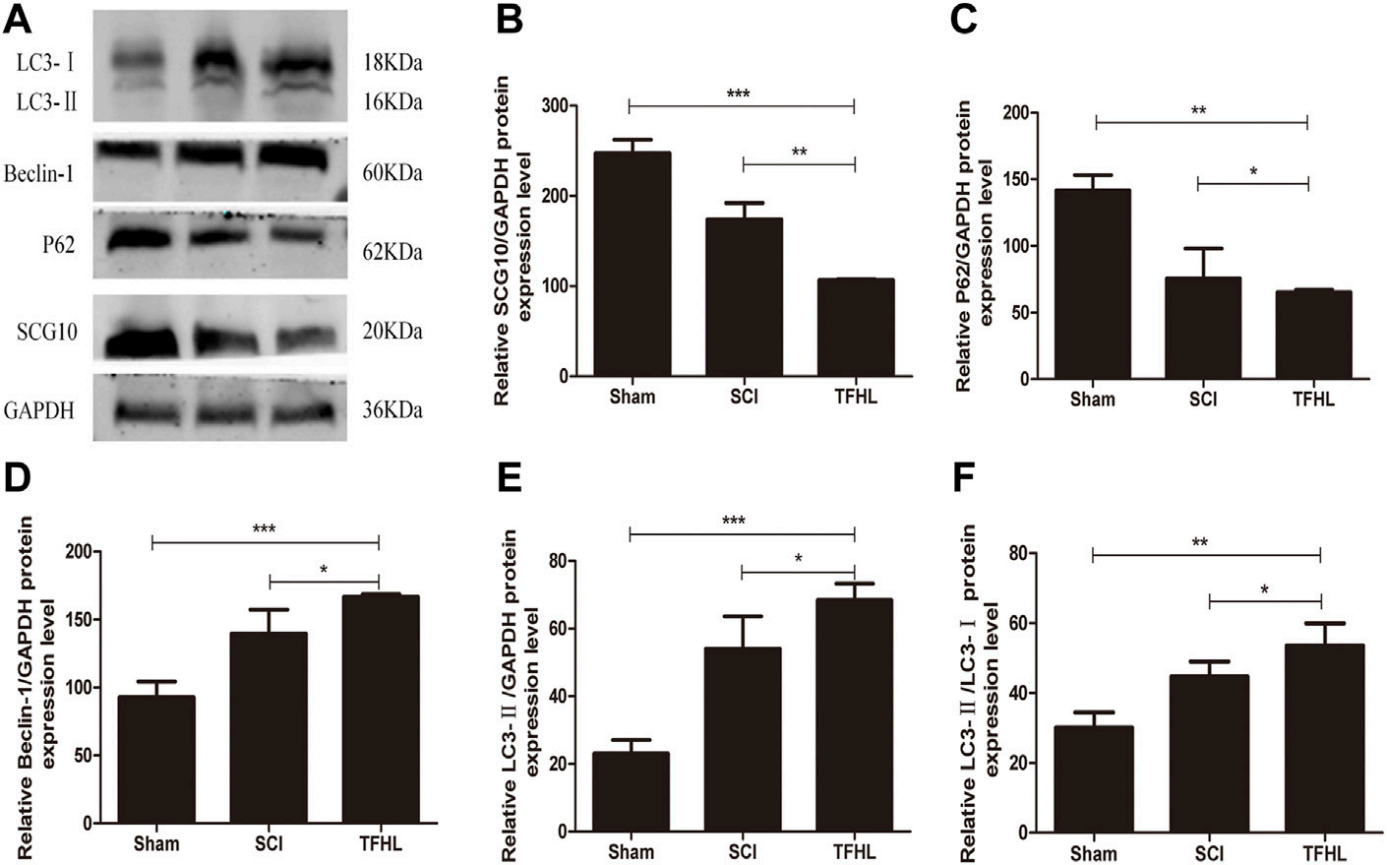
TFHL influences axons and autophagy related proteins expression in injured spinal cord tissues. **(A)** The expression of axon and autophagy-related protein in spinal cord tissues of rats in each group was assessed by Western blot assay. **(B–F)** The comparison of relative densitometry values between each group. All data are expressed as the mean ± SD. Differences among groups were determined with one-way ANOVA followed by a least-significant difference post hoc test. *n* = 6 per each group, *p < 0.05, **p < 0.01, ***p < 0.001.

The expression of autophagy-related proteins LC3, P62, Beclin-1, and microtubule destabilization-related protein SCG10 in damaged spinal cord tissues of rats following treatment with TFHL was detected by Western Blot. After homogenization with the internal reference protein GAPDH, the expression levels of LC3-Ⅱ, Beclin-1, and LC3-Ⅱ/LC3-Ⅰ in the spinal cord tissues of the SCI group were significantly increased compared with that of the Sham group, while SCG10 and P62 protein expression levels decreased (*p* < 0.05). Comparative to the SCI group, the THFL group had decreased P62 and SCG10 protein expression levels and increased LC3-Ⅱ, Beclin-1, and LC3-Ⅱ/LC3-Ⅰ protein expression levels (*p* < 0.05, Figure 7).

### Total flavonoids of hawthorn leaves promotes morphological repair of injured spinal motor neurons

**FIGURE 8:**
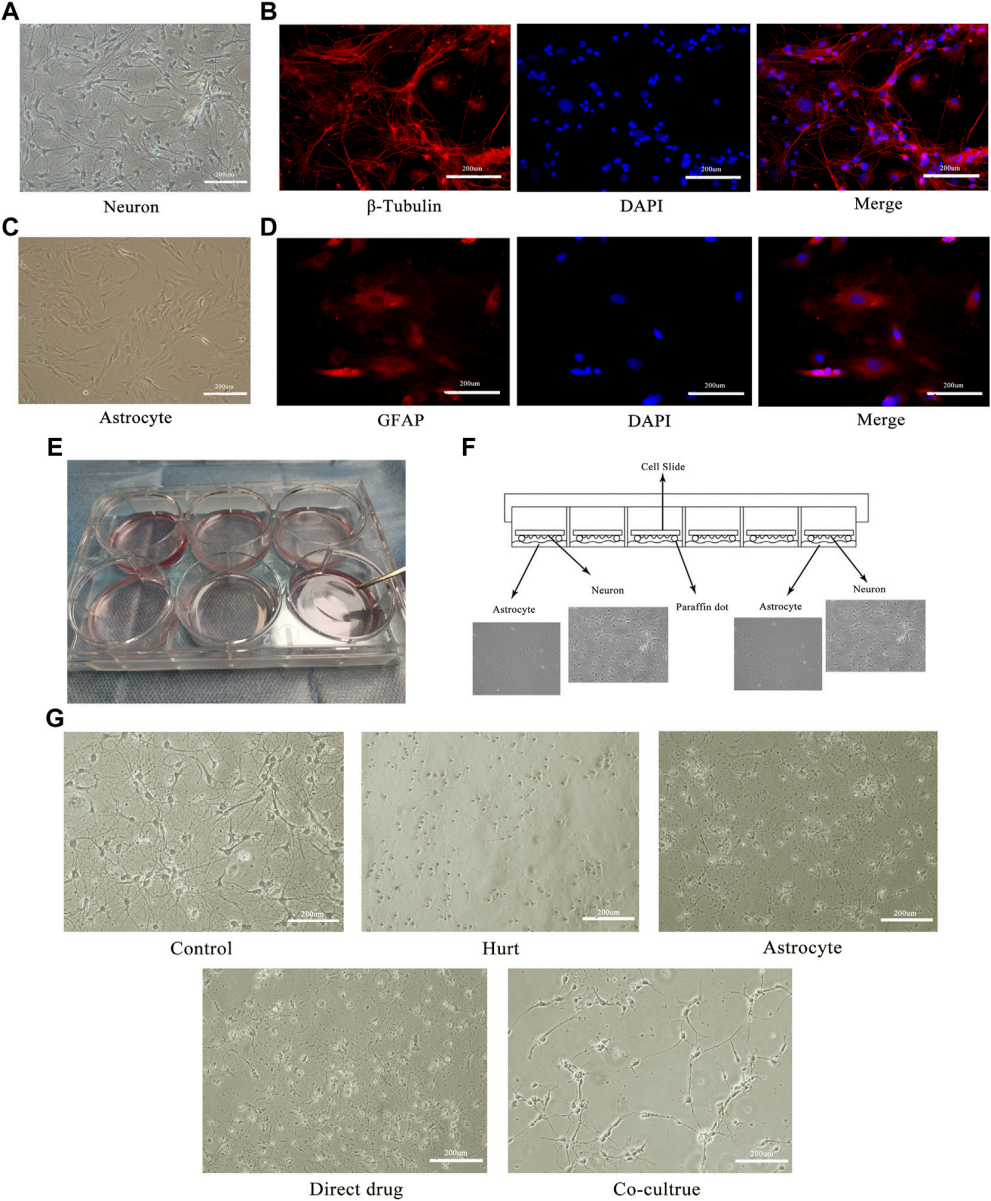
TFHL promotes morphological repair of injured SMNs. **(A)** The morphology of the motor neurons in the anterior horn of the spinal cord under a 20× optical microscope. **(B)** The immunofluorescence identification of spinal motor neurons with specific antibodies of β-Tubulin. **(C)** The morphology of astrocytes in the spinal cord under a 20× optical microscope. **(D)** The immunofluorescence identification of spinal cord astrocytes by GFAP specific antibody. **(E,F)** The physical image and schematic diagram of the cell co-culture model. **(G)** The changes of injured spinal motor neurons in each group after treatment with TFHL under a 20× optical microscope.

To further investigate the mechanisms of TFHL on the functional recovery of the spinal cord, *in vitro* experiments on SMNs were implemented. The morphological changes of SMNs after the intervention with TFHL on the cell co-culture model for 7 days were observed through an optical microscope. In the Control group, SMNs had a full-body, thick and long axons, and rich dendrites. After glutamate injury, the spinal cord motor neuron cell bodies in the Hurt group were significantly retracted, and axons and dendrites were broken. In contract to the Hurt group, SMNs in the Astrocyte group and Direct drug group had a certain degree of refraction, indicating that the neurons state had improved slightly. In the Co-culture group, some spinal motor neuron cell bodies became rounded and axons grew again, but there were fewer dendrites, indicating that the overall state of SMNs had been improved (Figure 8).

### Total flavonoids of hawthorn leaves facilitates microstructure and autophagosome change of injured spinal motor neurons

**FIGURE 9:**
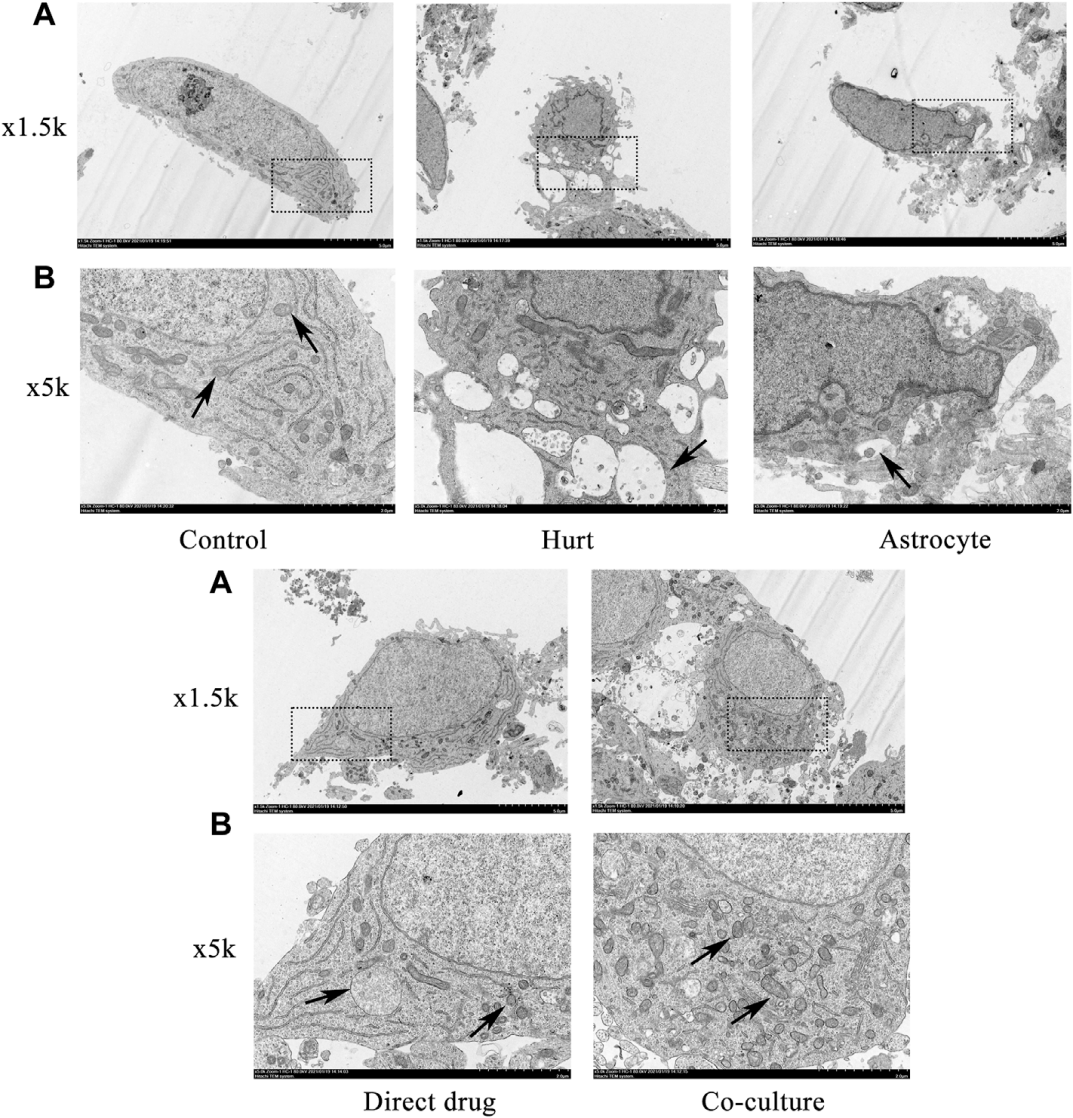
TFHL facilitates microstructure and autophagosome change of injured SMNs. Observation of microstructure and autophagosome change in SMNs of each group by transmission electron microscopy. **(A)** Under ×1.5 k electron microscope, the microstructure in SMNs of each group was observed, with scale bars = 5 μM. **(B)** Under a ×5 k electron microscope, the autophagosomes in SMNs were observed, with scale bars = 2 μM. The black dashed box is the observation site under the ×5 k magnification, the black arrow points to the autophagosome. SMNs: spinal motor neurons.

The microstructure and autophagosomes of the SMNs after the intervention with TFHL on the cell co-culture model for 7 days were observed by transmission electron microscope. Under ×1.5 k magnification, the nuclei of SMNs in the Control group were oval and had a regular shape. In the Hurt group, the nuclei of SMNs retracted, with irregular edges. In comparison with the Hurt group, the nucleus morphology of SMNs in the Astrocyte group, Direct drug group, and Co-culture group was restored, in which the Co-culture group was the best. Under ×5 k magnification, autophagosomes can be clearly seen in SMNs of the Control group. When the neurons were injured, there were many autophagosomes of SMNs that are working, which wrapped the damaged organelles and macromolecular substances in the Hurt group. Autophagosomes in a working state can also be seen in SMNs in the Astrocyte group, Direct drug group, and Co-culture group, and the number of autophagosomes in the Co-culture group had increased significantly (Figure 9).

### Total flavonoids of hawthorn leaves conduces axonal regeneration of injured spinal motor neurons

**FIGURE 10:**
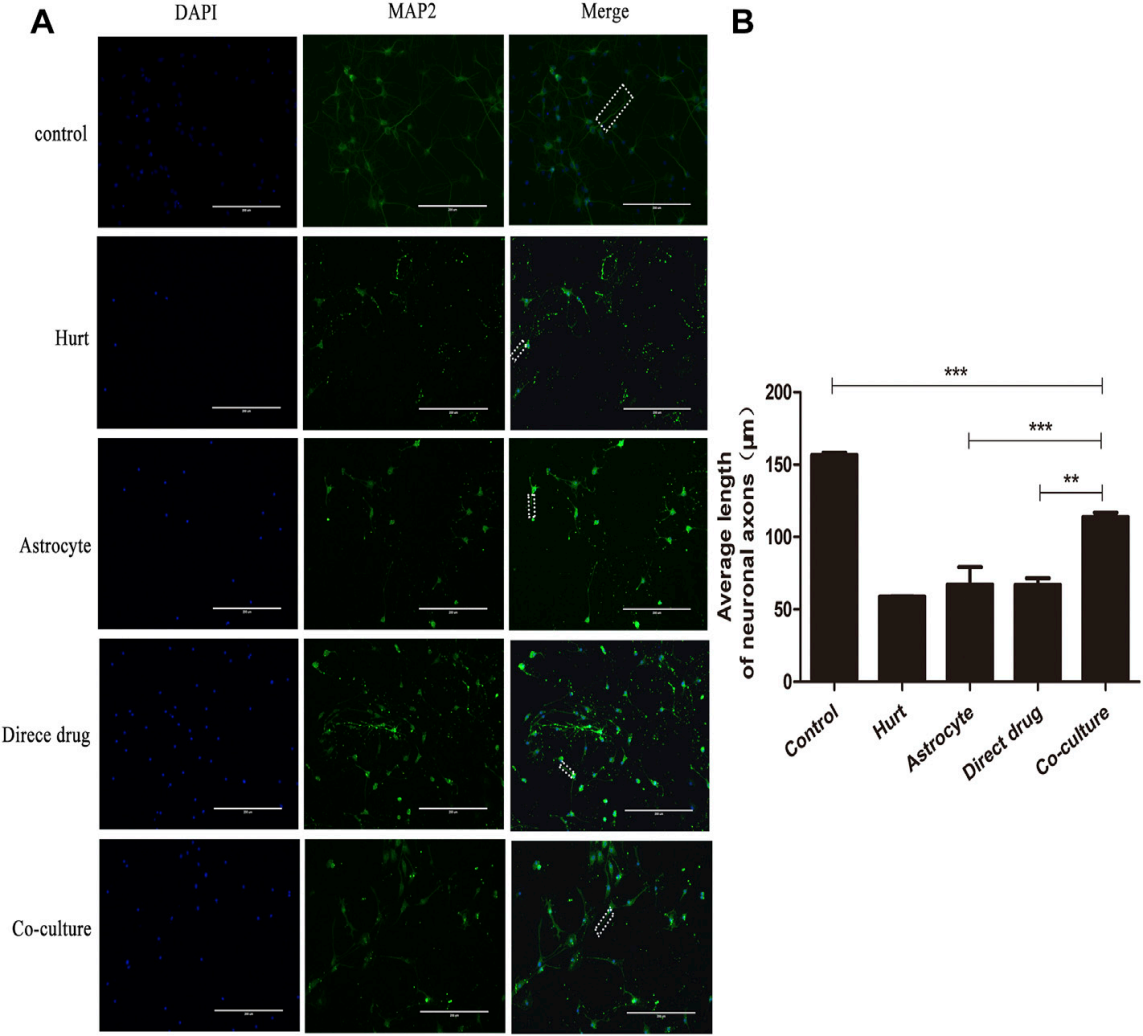
TFHL conduces axonal regeneration of injured SMNs. **(A)** Under ×20 magnification, the regeneration of spinal motor neuron axons in each group was observed by MAP2 Immunofluorescence, scale bars = 200 μM. DAPI is nuclear staining with blue fluorescence; MAP2 is neuron tubulin with green fluorescence; Merge is a composite image of the former two. **(B)** The comparison of the average length of axon regeneration of SMNs in each group. The white wireframe shows axons. All data are expressed as the mean ± SD. Differences among groups were determined with one-way ANOVA followed by a least-significant difference post hoc test. *p < 0.05, **p < 0.01, ***p < 0.001. SMNs: spinal motor neurons.

The axon regeneration of SMNs after the intervention with TFHL on the cell co-culture model for 7 days was observed by MAP2 immunofluorescence staining. Under ×20 magnification, the axons of SMNs were thick and long, and the dendrites were intertwined into a network in the Control group. After glutamate damaged neurons, only the neuron cell bodies with green fluorescence were seen, and its axons and dendrites were difficult to see in the Hurt group. For the Astrocyte group, Direct drug group, and Co-culture group, the growth of SMNs axons and dendrites was slightly better than that of the Hurt group (Figure 10A). Comparing the average axon length of SMNs in each group found that the average axon length of SMNs in the Hurt group was significantly different from that in the Control group (*p* < 0.001). As opposed to the Hurt group, there was no significant difference in the average axon length of SMNs between the Astrocyte group and the Direct drug group (*p* > 0.05). The average axon length of SMNs in the Co-culture group was significantly different from the first three groups, indicating that the average axon length of SMNs in the Co-culture group was the longest and had the best regeneration effect (*p* < 0.001, Figure 10B).

### Total flavonoids of hawthorn leaves enhances autophagy of injured spinal motor neurons

**FIGURE 11:**
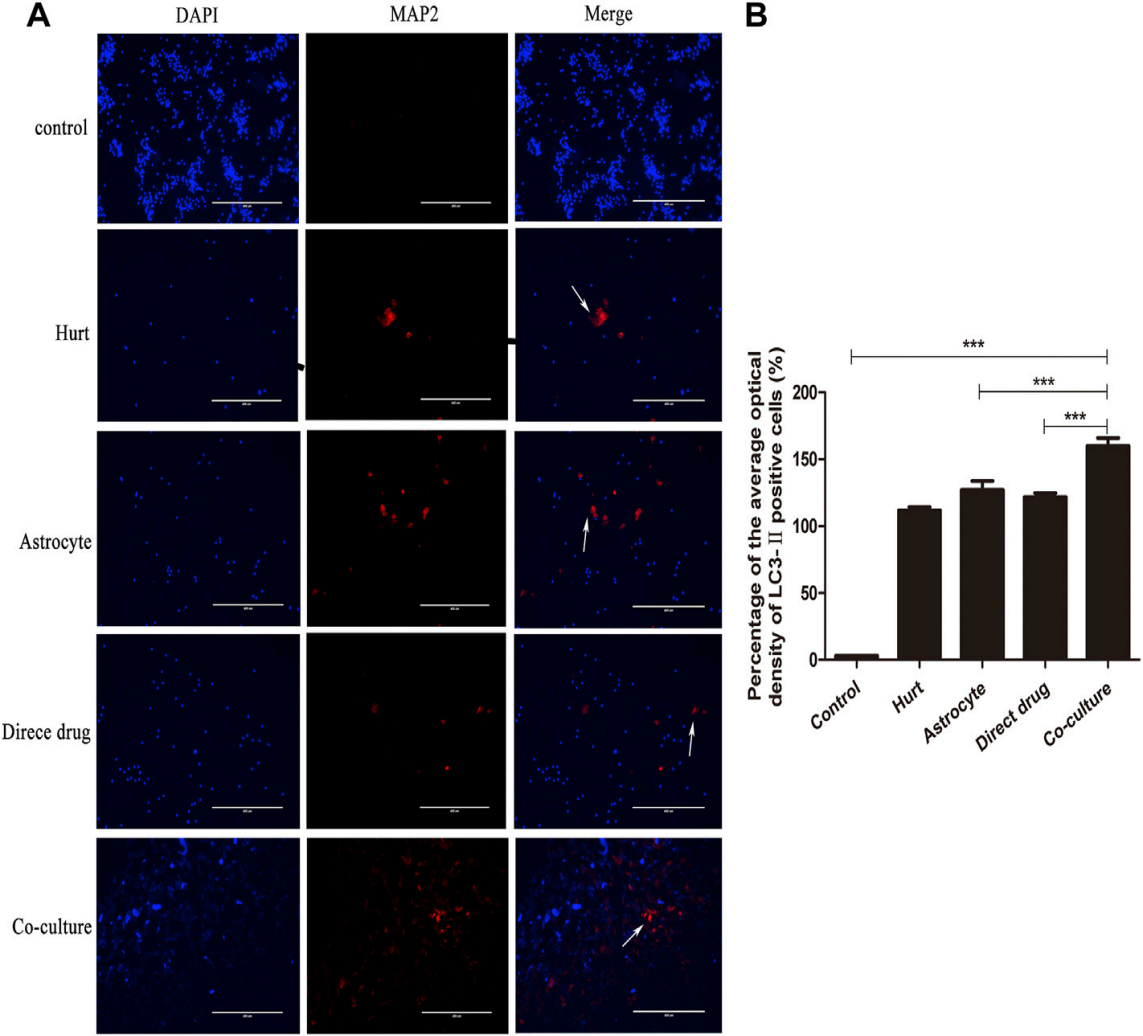
TFHL enhances autophagy of injured SMNs. **(A)** Under ×10 magnification, the autophagy expression in SMNs of each group was observed by LC3-ⅡImmunofluorescence, with scale bars = 400 μM. DAPI was a nuclear stain with blue fluorescence; LC3-Ⅱ was a related protein with red fluorescence; Merge was a composite image of the former two. **(B)** The comparison of the average optical density ratio of LC3-Ⅱ in each group. All data are expressed as the mean ± SD. Differences among groups were determined with one-way ANOVA followed by a least-significant difference post hoc test. *p < 0.05, **p < 0.01, ***p < 0.001. SMNs: spinal motor neurons.

The autophagy expression of SMNs after the intervention with TFHL on the cell co-culture model for 7 days was observed by LC3-Ⅱ immunofluorescence staining. Under ×10 magnification, the positive LC3-Ⅱ with red fluorescence can be observed in the Hurt group, Astrocyte group, Direct drug group, and Co-culture group (Figure 11A). Comparing the average optical density value of LC3-Ⅱ in SMNs of each group found that the average optical density of LC3-Ⅱ in spinal motor neurons in the Hurt group was significantly higher (*p* < 0.001). While the average optical density of LC3-Ⅱ in SMNs in the Astrocyte group and the Direct drug group had no significant difference (*p* > 0.05). The average optical density value of LC3-Ⅱ in the Co-culture group increased the most significantly compared with that of the first three groups, indicating that the Co-culture group had the highest level of autophagy (*p* < 0.001, Figure 11B).

### Total flavonoids of hawthorn leaves promotes the secretion of neurotrophic factors by astrocytes

**FIGURE 12:**
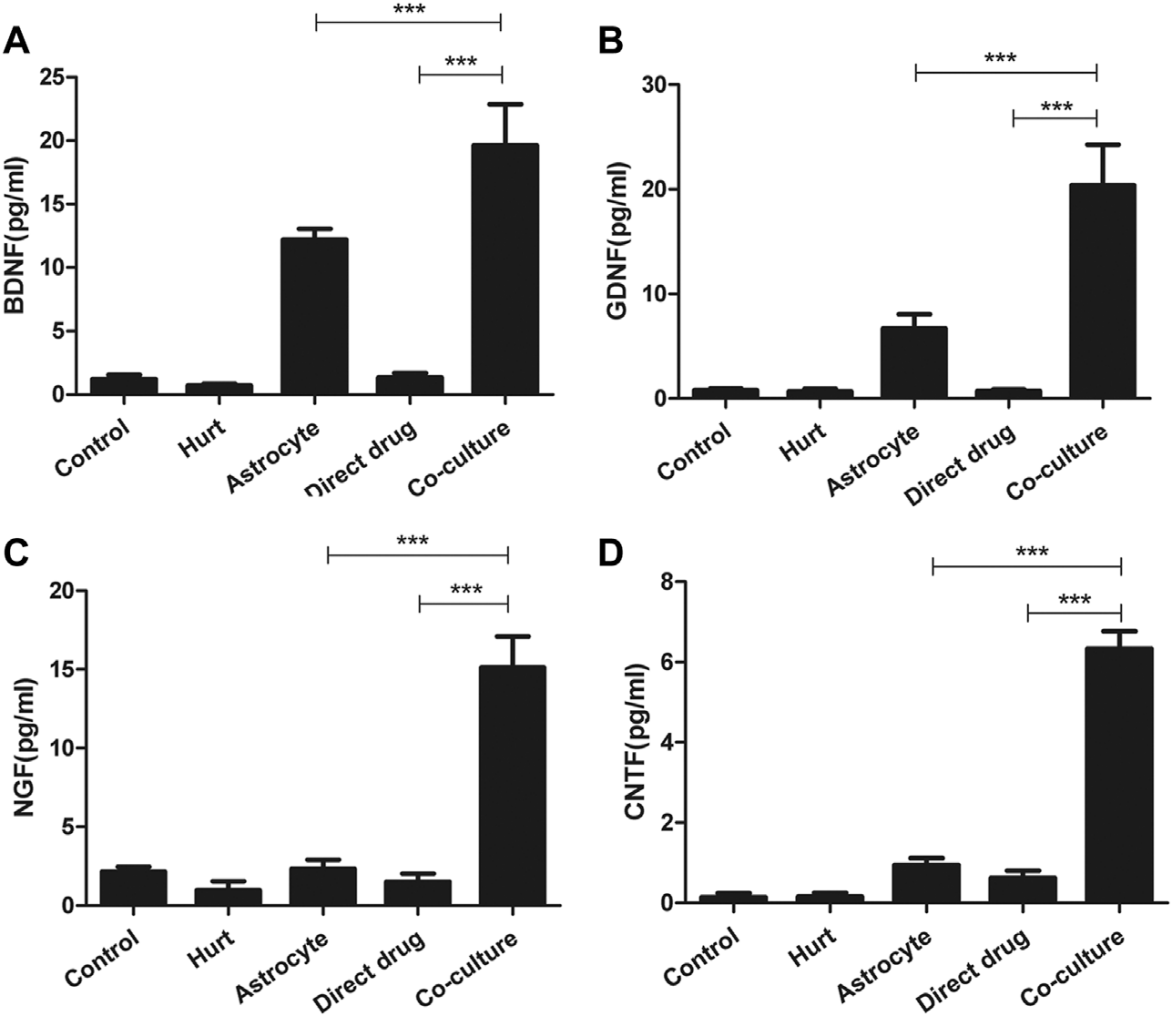
TFHL promotes secretion of neurotrophic factors by astrocytes. **(A–D)** The secretion of BDNF, GDNF, NGF, and CNTF in each group was detected by ELISA. The comparison of the contents of BDNF, GDNF, NGF, and CNTF in the culture medium of each group. All data are expressed as the mean ± SD. Differences among groups were determined with one-way ANOVA followed by a least-significant difference post hoc test. *p < 0.05, **p < 0.01, ***p < 0.001.

The secretion of BDNF, GDNF, NGF, and CNTF in each group after the intervention with TFHL on the cell co-culture model for 7 days was detected by ELISA. The results found that a small number of neurotrophic factors such as BDNF, GDNF, NGF, and CNTF can be detected in the Control group, the Hurt group, and Direct drug group, but there was no statistical significance (*p* > 0.05). The Astrocyte group showed statistically significant levels of BDNF, GDNF, and CNTF in comparison with the Hurt group (*p* < 0.05). Notably, In contrast to the first four groups, the content of BDNF, GDNF, NGF, and CNTF in the Co-culture group was significantly increased, indicating that TFHL increased the content of neurotrophic factors after intervening in the co-culture model (*p* < 0.001, Figure 12).

### Total flavonoids of hawthorn leaves influences protein expression linked to axons and autophagy in injured spinal motor neurons

**FIGURE 13:**
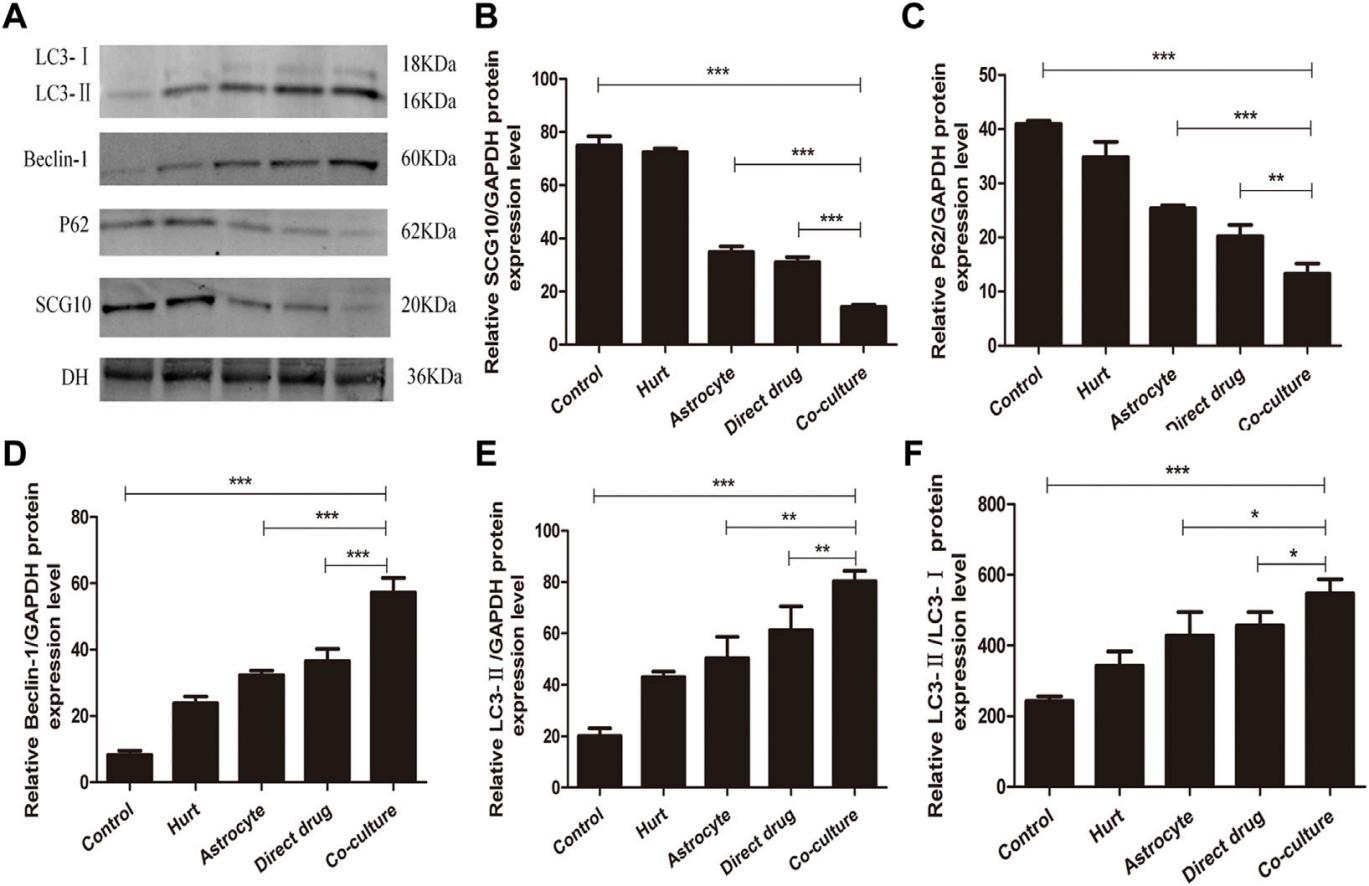
TFHL influences axons and autophagy related proteins expression in injured SMNs. **(A)** The expression of axon and autophagy-related protein in SMNs of each group was assessed by Western blot assay. **(B–F)** The comparison of relative densitometry values between each group. All data are expressed as the mean ± SD. Differences among groups were determined with one-way ANOVA followed by a least-significant difference post hoc test. *p < 0.05, **p < 0.01, ***p < 0.001. SMNs: spinal motor neurons.

The expression of autophagy-related proteins LC3, P62, Beclin-1, and microtubule destabilization-related protein SCG10 in SMNs after intervention with TFHL was measured by Western Blot. After homogenization with the internal reference protein GAPDH, a significant increase was found in LC3-II, Beclin-1, and LC3-II/LC3-I expression in SMNs of the Hurt group when compared to the Control group (*p* < 0.05), while the expression level of P62 protein decreased (*p* < 0.01), the expression of SCG10 protein was not statistically significant (*p* > 0.05). In comparison with the Hurt group, the P62 and SCG10 protein expression levels of SMNs in the Astrocyte group and Direct drug group decreased, and the protein expression levels of LC3-Ⅱ/LC3-Ⅰ and Beclin-1 increased (*p* < 0.05). However, the expression of LC3-Ⅱ protein was not statistically significant between the Hurt group and Astrocyte group, and there was no significant difference between the Astrocyte group and the Direct drug group (*p* > 0.05). In the Co-culture group, the protein expression level was significantly different from the Hurt group, Astrocyte group, and Direct drug group, indicating that the protein expression levels linked to axons and autophagy in SMNs changed significantly (*p* < 0.05, Figure 13).

## Discussion

In this study, we demonstrated that TFHL improved neuromotor function recovery after SCI. TFHL also promoted injured spinal cord tissue repair, while suppressing cell apoptosis, and improving the functional status of neurons in the spinal cord tissues. We further explored the reason for the functional recovery of the injured spinal cord and rescued neurons and found that TFHL enhanced autophagy levels and related protein expression, while promot axonal regeneration. Our *in vitro* study provided direct evidence that TFHL protected damaged SMNs and enhanced autophagy in SMNs, which probably was one of the key causes to promote injured SMNs repair. Additionally, increased neurotrophic factor secretion was found in the co-culture model of astrocyte and SMNs after intervention with TFHL, indicting TFHL maybe improved surrounding conditions around SMNs.

TFHL has a rich variety and high content of flavonoids. In our study, various flavonoids in TFHL were analyzed by Q-Orbitrap high-resolution LC-MS technology, in which some flavonoids were more obvious, including Eriodictyol, Rhoifolin, Catechin, Quercetin, Isorhamnetin, Astragalin, Genistein, Naringenin, Kaempferol-7-O-glucoside, Luteolin, 3-Methoxy-5,7,3',4'-tetrahydroxy-flavone, (-)-Epigallocatechin, Taxifolin, Rutin, 7-Hydroxy-2-(4-hydroxyphenyl)-4-oxo-3,4-dihydro-2H-chromen-5-yl β-D-glucopyranoside, Quercetin 3β-D-glucoside, 4H-1-Benzopyran-4-one, 6-β-D-glucopyranosyl-2,3-dihydro-5,7-dihydroxy-2-(4-hydroxyphenyl)-,(S)-, (1S)-1,5-Anhydro-2-O-(6-deoxy-α-L-mannopyranosyl)-1-[5,7-dihydroxy-2-(4-hydroxyphenyl)-4-oxo-4H-chromen-6-, etc. It was reported that flavonoids have therapeutic potential for SCI, comprising anti-inflammatory properties, anti-oxidant properties, anti-apoptotic properties, neuroprotective properties, and so on (Zhang et al., 2017). Currently, the research on flavonoids treated SCI has made certain progress. Related research has shown that (-)-Epigallocatechin can promote recovery after SCI by reducing inflammation and neuronal apoptosis, while significantly improving the recovery of motor function (Lu et al., 2020). Quercetin can promote motor function recovery and axonal regeneration through inducting autophagy and preventing necroptosis of oligodendrocytes after SCI (Fan et al., 2019; Wang et al., 2021). Naringenin can exert neuroprotective effects by inducing neurotrophic factors and suppressing the apoptotic pathways (Abbaszadeh et al., 2020). Rutin can repress the cellular inflammatory response after SCI through suppressing the activation of TGF-β/Smad pathway (Yao et al., 2021). Luteolin can suppress oxidative stress and preserve mitochondrial function after SCI through signaling pathways involving Nrf2 activation and downstream gene expression (Fu et al., 2021). Isorhamnetin can promote functional recovery after SCI by abating oxidative stress and modulating M2 macrophages/microglia polarization (Chen et al., 2021). Astragalin can protect against SCI through attenuating oxidative stress-induced necroptosis (Sun et al., 2021). Although a single flavonoid has been proven to be effective for SCI, there are still few studies on the efficacy of total flavonoids for SCI from the perspective of multi-component and multi-target. Therefore, exploring the effect of TFHL on SCI remains certain research value. Our previous study displayed that TFHL can promote motor function recovery and reduce spinal cord tissue damage while inhibiting apoptosis in spinal cord tissue after SCI (Zhang et al., 2021). Our current study further focuses on the protection of TFHL on SMNs after SCI *in vivo* and *in vitro* experiments.

SMNs are one type of multilevel neuron mainly distributed in the anterior horn of spinal cord and can form the motor end-plate with skeletal muscle fibers through the extension of axons to innervate skeletal muscles and control voluntary movements of the body (Yang et al., 2015). Thus, SMNs play a vital role in the motor function of the spinal cord. It is proven that SCI is prone to induce secondary death of SMNs, in which apoptosis is one of the key factors leading to SMNs death (Shi et al., 2021). Apoptosis is regulated by multiple related genes and proteins and is also affected by the extracellular environment (D’arcy, 2019). We found that the inner environment in spinal cord tissues was significantly improved after treatment with TFHL by observation of HE staining and transmission electron microscopy, probably providing a favorable condition for SMNs survival and relieving apoptosis in the spinal cord tissue. Meanwhile, the improved function status of SMNs in spinal cord tissue treated with TFHL was found by Nissl staining. Nissl bodies are often used as markers of neuronal function. There are abundant Nissl bodies in neurons with strong metabolic function, while Nissl bodies are reduced, disintegrated, or even disappear in damaged neurons, and during the recovery, Nissl bodies can reappear, increase, and reach normal levels (Niu et al., 2015). Consequently, the results from our *in vivo* experiments can preliminarily demonstrate that TFHL has protective effects on injured SMNs in the spinal cord tissue.

Given that there are various cells and other complicated compositions in spinal cord tissue, we can’t get accurate results by relying only on *in vivo* experiments. Therefore, the co-culture system of astrocytes and SMNs proposed by Pamela J *et al* from Washington University was constructed (Roque and Costa, 2017). We noticed that TFHL can promote injured SMNs repair, in which the recovery of injured SMNs in the Co-culture group was the best compared with other groups, suggesting that astrocytes may be an advantage factor for promoting injured SMNs repair. Further, we found that there was higher content of neurotrophic factors, a class of functional proteins acted on neurons, comprising BDNF, GDNF, NGF, and CNTF in the Co-culture group. It is widely accepted that astrocytes are the most abundant glial cell type in the central nervous system and have been implicated in the mechanical and metabolic support of neurons (Lukovic et al., 2015). In the early or specific stage of injury, astrocytes exert neuroprotective effects through secreting neurotrophic factors (Kisucka et al., 2021). Meanwhile, a diverse body of literature supports that under certain conditions, the tissue surrounding an injured neuron can release a series of endogenous factors to promote its repair and regeneration, in which neurotrophic factors are major functional endogenous factors (Dremencov et al., 2021). Different neurotrophic factors exert specific functions. BDNF can enhance the survival rate of injured neurons in spinal cord tissue, and accelerate the repair and regeneration of injured spinal neurons (Phillips, 2017). GDNF, a member of the transforming growth factor B superfamily, is closely associated with the proliferation, differentiation, and migration of neurons (Tao et al., 2019). NGF, widely distributed in the brain, spinal cord, and surrounding tissues, not only regulates the number of neurons but affects the growth of axons and dendrites (Ciafrè et al., 2020). CNTF, a neurotrophic factor found firstly to maintain the survival and growth of neurons *in vivo* and *in vitro*, plays a crucial role in the restorative process of neurons (Jia et al., 2019). Related studies also reveal that flavonoids (Quercetin, (-)-Epigallocatechin, etc.), can promote astrocytes to secrete neurotrophic factors (Wang Y. et al., 2018). Consistent with these findings, we speculated that TFHL can facilitate astrocytes to secrete neurotrophic factors, promoting injured SMNs repair.

The functional recovery of injured SMNs largely relies on the regeneration of axons (Godinho et al., 2020). In the present study, we found that the axons of injured SMNs were reconstructed after treatment with TFHL. Furthermore, the decreased protein expression of superior cervical ganglia neural-specific protein 10 (SCG10) further demonstrated the ability of TFHL to promote axonal regeneration. SCG10, a microtubule destabilizing factor specifically expressed in neurons, affects the growth of axons through exerting the functions of microtubule depolymerization and regulating the dynamic changes of microtubules (Shin et al., 2012). It is reported that autophagy can induce microtubule stability to promote the regeneration of axons through down-regulation of the SCG10 expression level (Shin et al., 2014). It is worth mentioning that we found the autophagy level increased following SCI, while the ascension of autophagy level was more obvious after treatment with TFHL, suggesting that TFHL possibly enhance autophagy. Additionally, we further found that the expression of autophagy-related proteins Beclin-1, LC3-II, and LC3-II/LC3-I increased, while P62 protein expression decreased after treatment with TFHL from the results *in vivo* and *in vitro* experiments, highly indicating that the enhancement of autophagy expression after treating with TFHL, in which Beclin-1, LC3-I, LC3-II, and P62 are key marker genes involved autophagy regulation (Levine and Kroemer, 2019). Various studies have shown that autophagy is an essential degradation pathway involved in the secondary phase of SCI (Kanno et al., 2009). Enhanced autophagy may be protective after moderate injury, while the inhibition of autophagy after severe injury may lead to neuronal cell death (Zhou et al., 2017). Although the precise role of autophagy, whether detrimental or beneficial, in preclinical models of neurotrauma, is still controversial, combined with our whole findings, we speculated that TFHL can enhance autophagy levels to promote injured SMNs restoration after SCI.

In our study, several factors could limit the extent to which the results can be generalized. First, in the experimental design stage, there was no gold standard drug for treating SCI clinically, so this study did not set up a positive control group to compare the efficacy of TFHL and other drugs. Moreover, we only detected the content of neurotrophic factors *in vitro* experiment, probably leading to less persuasive results. Second, we mainly focused on the effect of TFHL on SMNs, a highly differentiated terminal cell with the characteristics of short survival time and high culture requirements, limiting the depth of research.

In conclusion, TFHL may exert neuroprotective effects on SMNs by enhancing autophagy levels to promote the recovery of neuromotor function of the spinal cord after SCI. Our findings can provide some reference value for the research on flavonoids or motor neuron.

## Data Availability

The original contributions presented in the study are included in the article/Supplementary Material, further inquiries can be directed to the corresponding authors.
