# Supplementary material for: Total flavonoids of hawthorn leaves protect spinal motor neurons via promotion of autophagy after spinal cord injury
Source: Front Pharmacol. 2022 Aug 22;13:925568. doi: 10.3389/fphar.2022.925568 (PMC9441667; doi:10.3389/fphar.2022.925568)
Supplement: Supplementary file 1 [file DataSheet1.PDF]

The Composition of Total Flavonoids in Hawthorn Leaves

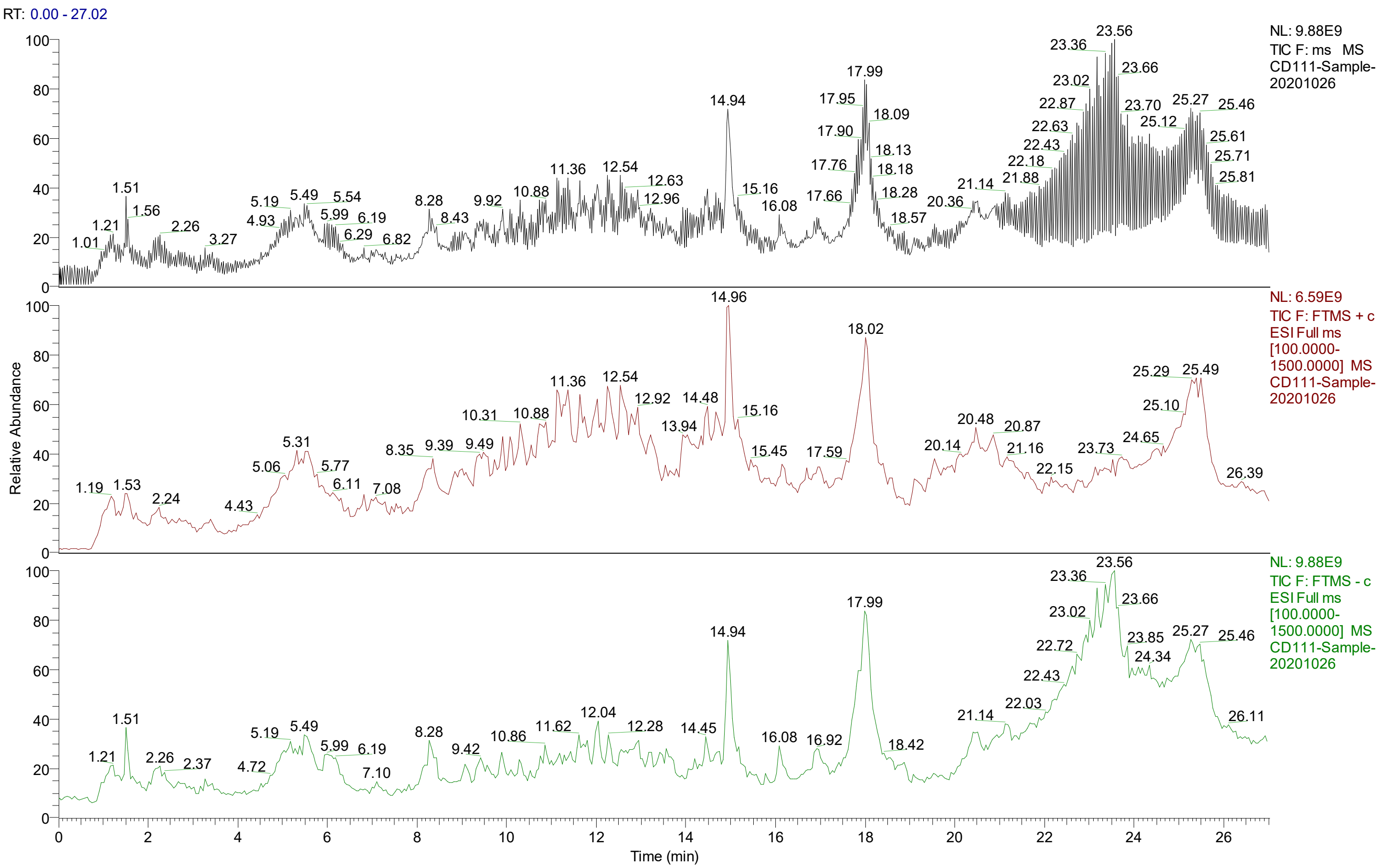

Figure 1 The total ion current spectrum of TFHL

The mass spectrometry data of the TFHL was collected by Q-Orbitrap high-resolution LC-MS technology. According to chromatographic retention time, primary and secondary mass spectrometry information, after processing by metabolomics data analysis software CD2.1, search and compare with the database (mzCloud, mzVault, ChemSpider) to obtain the corresponding substance composition. In the figure 1, the black represents the superposition of the positive and negative total ion current maps in the first column, the red represents the total ion current diagram in positive ion mode in the second column, and the green is the total ion current map in the negative ion mode in the third column. In the mzCloud best match, there are 19 compounds with a comprehensive score greater than 80, as follows:

Compounds

03-Nov-2020 11:15

Structure	Name	Formula	FISh Coverage	RT [min]	Molecular Weight	mzCloud Best Match
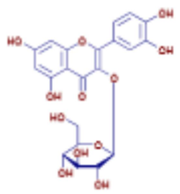	Quercetin-3β-D-glucoside	C21 H20 O12	30.43	5.23	464.0956	95.0

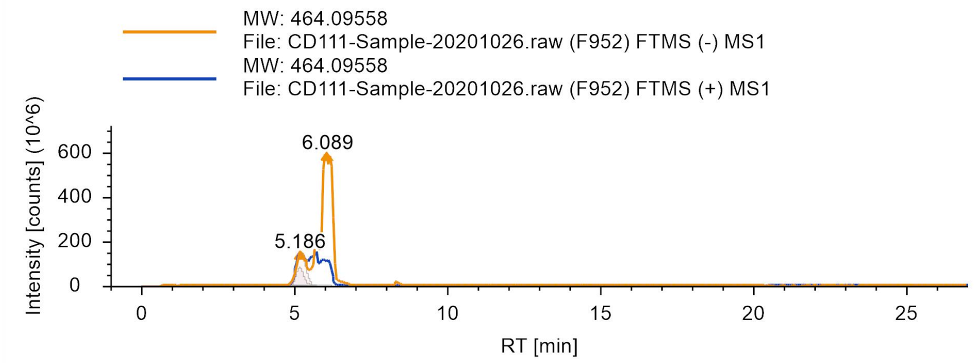

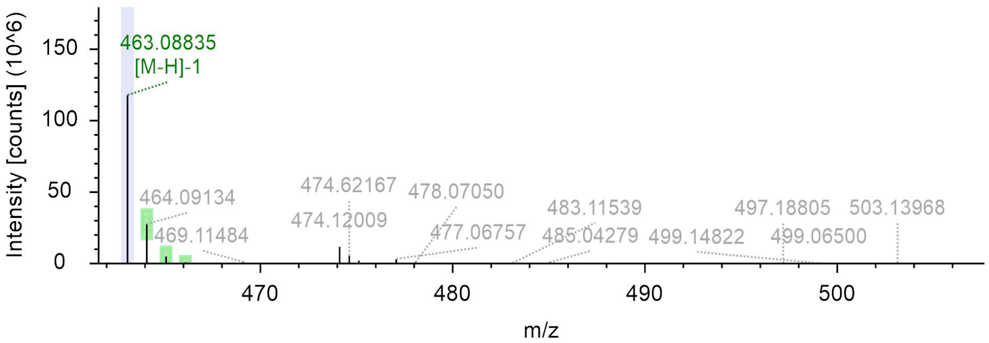

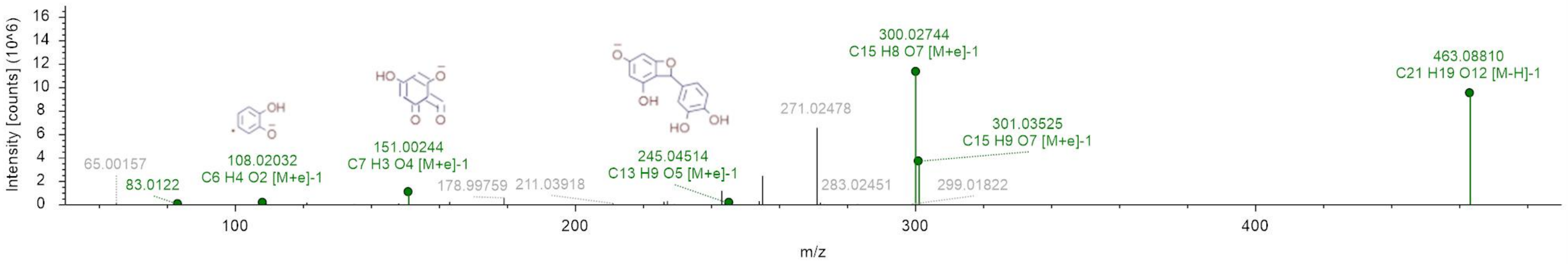

© Reported with Compound Discoverer 2.1

Compounds

03-Nov-2020 11:15

Structure	Name	Formula	FISh Coverage	RT [min]	Molecular Weight	mzCloud Best Match
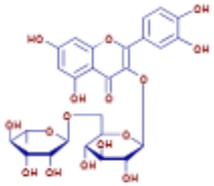	Rutin	C27 H30 O16		6.32	610.1533	94.1

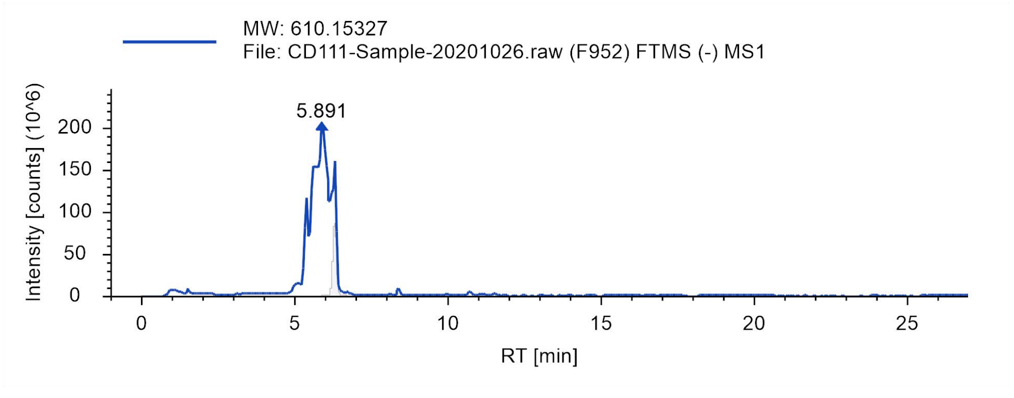

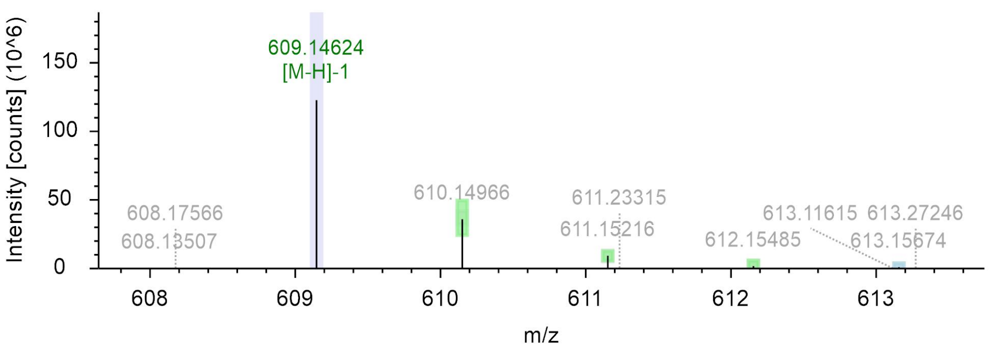

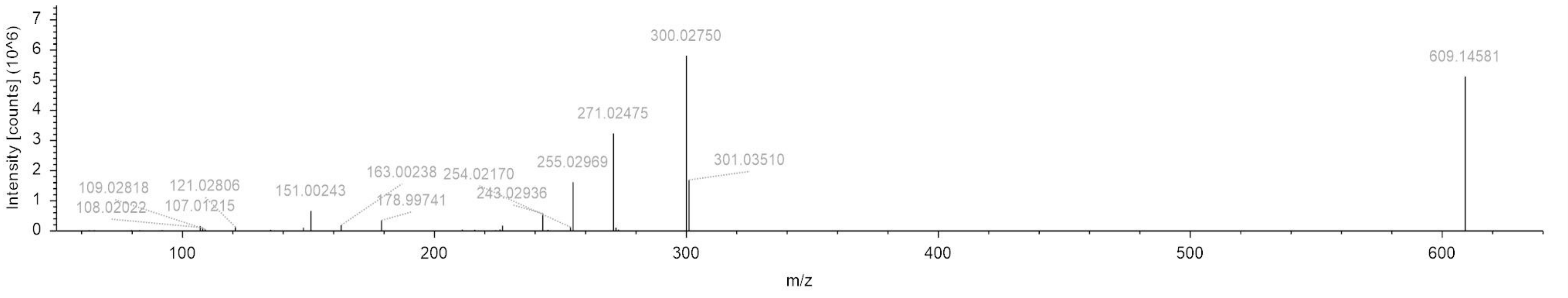

© Reported with Compound Discoverer 2.1

Compounds

03-Nov-2020 11:15

Structure	Name	Formula	FISh Coverage	RT [min]	Molecular Weight	mzCloud Best Match
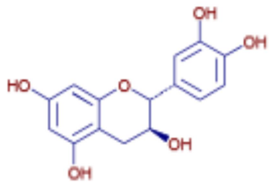	Catechin	C15 H14 O6		8.29	290.0787	93.7

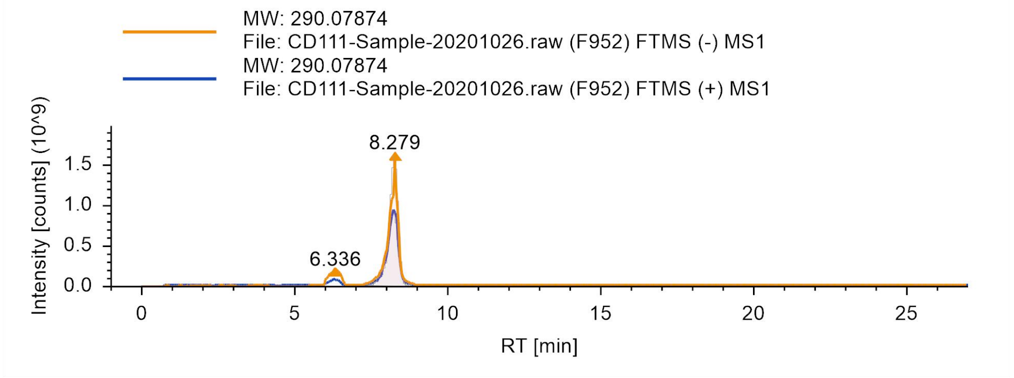

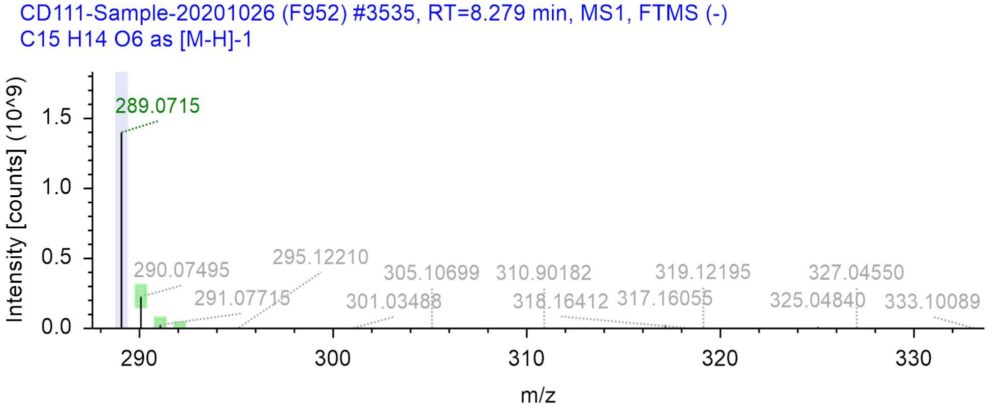

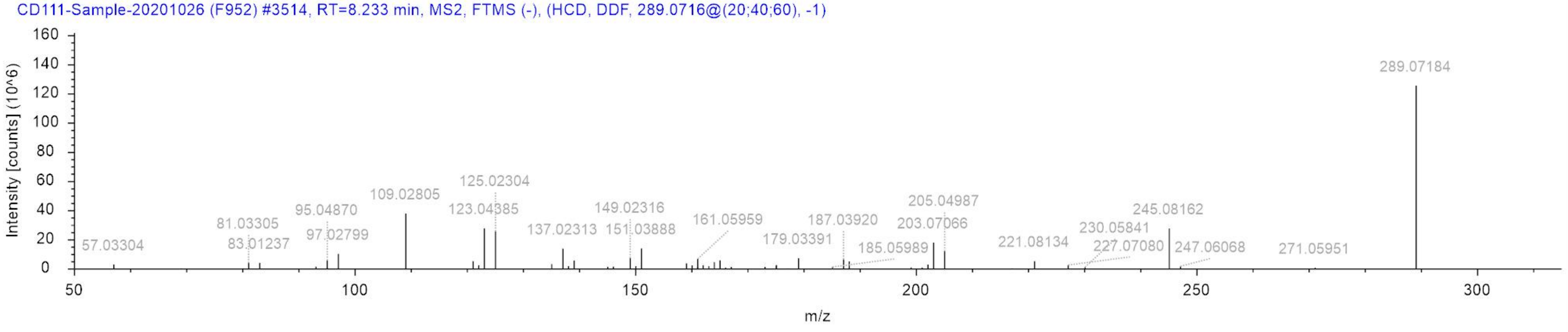

© Reported with Compound Discoverer 2.1

Compounds

03-Nov-2020 11:15

Structure	Name	Formula	FISh Coverage	RT [min]	Molecular Weight	mzCloud Best Match
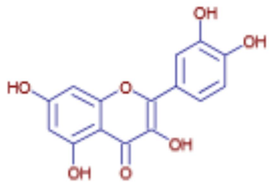	Quercetin	C15 H10 O7		6.59	302.0429	92.7

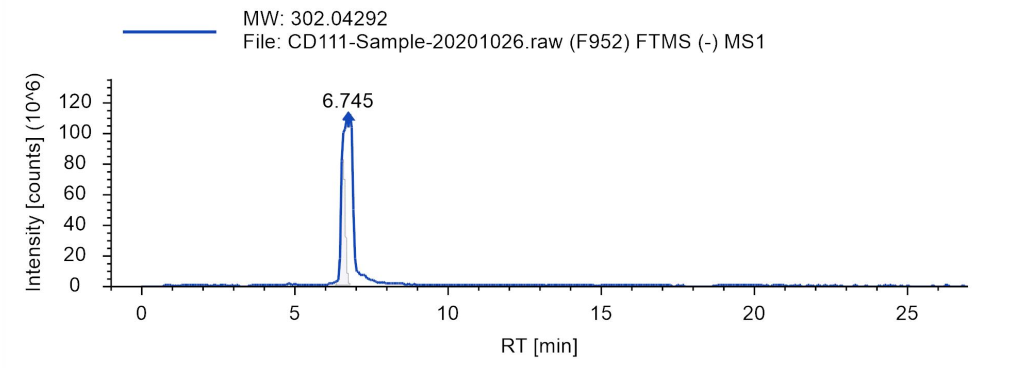

CD111-Sample-20201026 (F952) #2809, RT=6.591 min, MS1, FTMS (-)
C15 H10 O7 as [M-H]-1

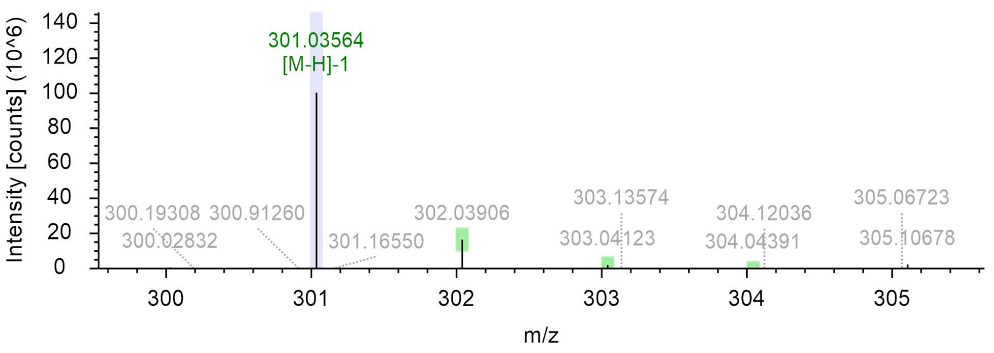

CD111-Sample-20201026 (F952) #2832, RT=6.648 min, MS2, FTMS (-), (HCD, DDF, 301.0356@(20;40;60), -1)

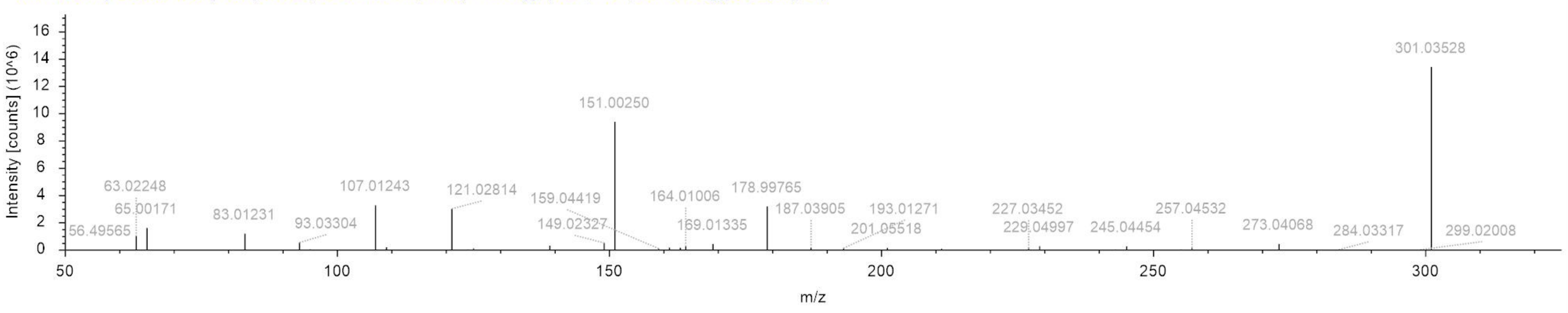

© Reported with Compound Discoverer 2.1

Compounds

03-Nov-2020 11:15

Structure	Name	Formula	FISh Coverage	RT [min]	Molecular Weight	mzCloud Best Match
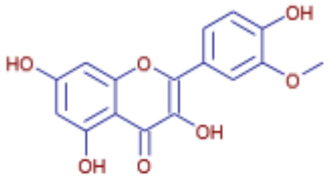	Isorhamnetin	C16 H12 O7		6.69	316.0581	90.1

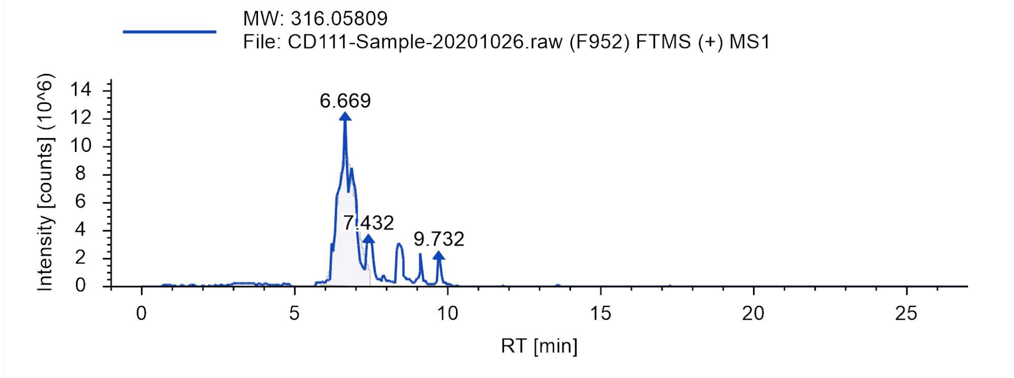

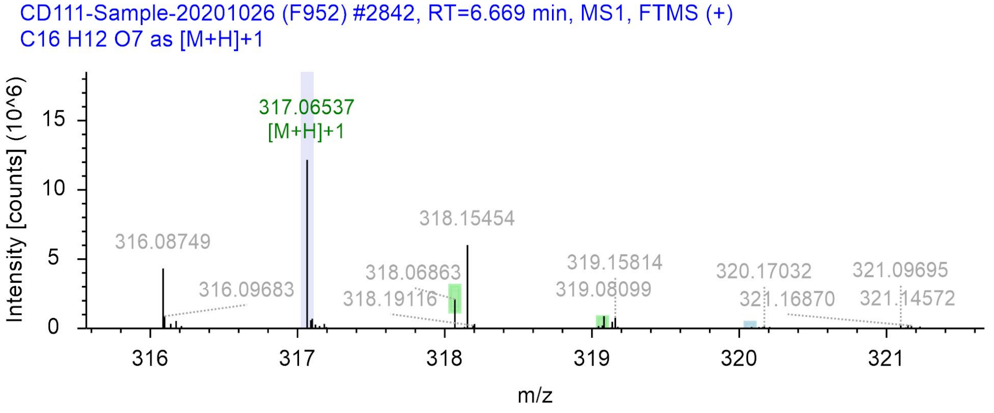

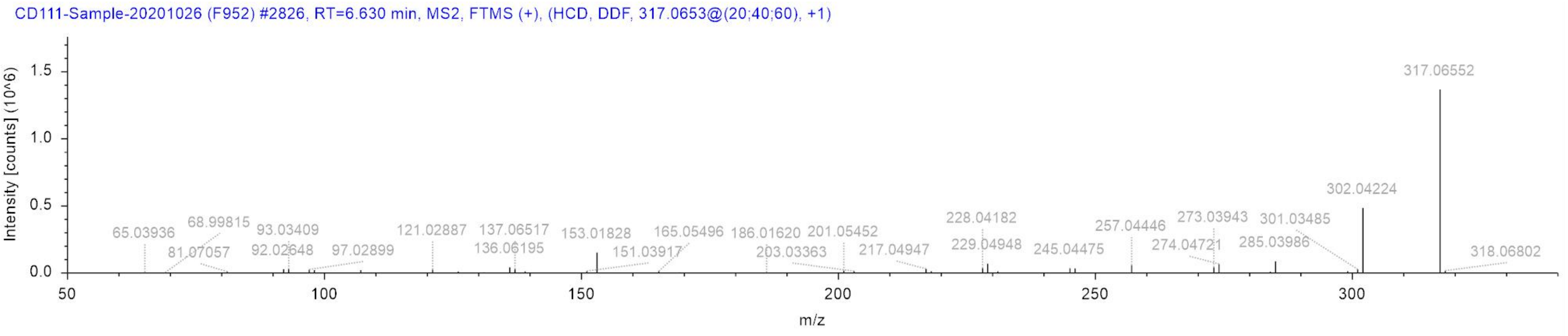

© Reported with Compound Discoverer 2.1

Compounds

03-Nov-2020 11:15

Structure	Name	Formula	FISh Coverage	RT [min]	Molecular Weight	mzCloud Best Match
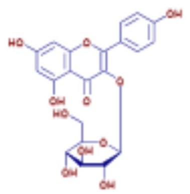	Astragalin	C21 H20 O11		6.64	448.1006	89.6

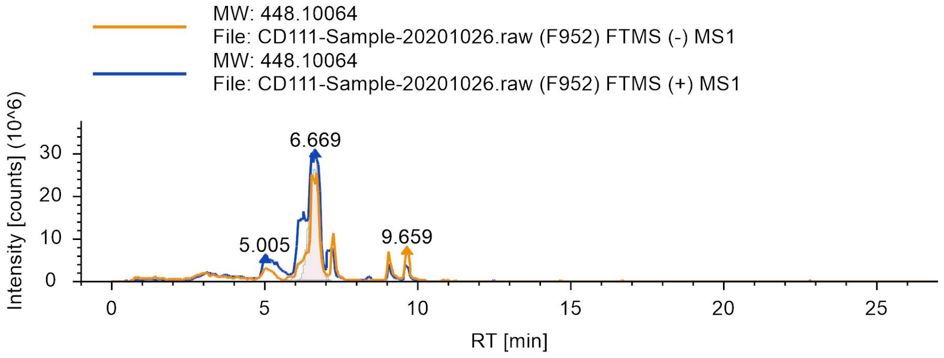

CD111-Sample-20201026 (F952) #2820, RT=6.618 min, MS1, FTMS (+)
C21 H20 O11 as [M+H]+1

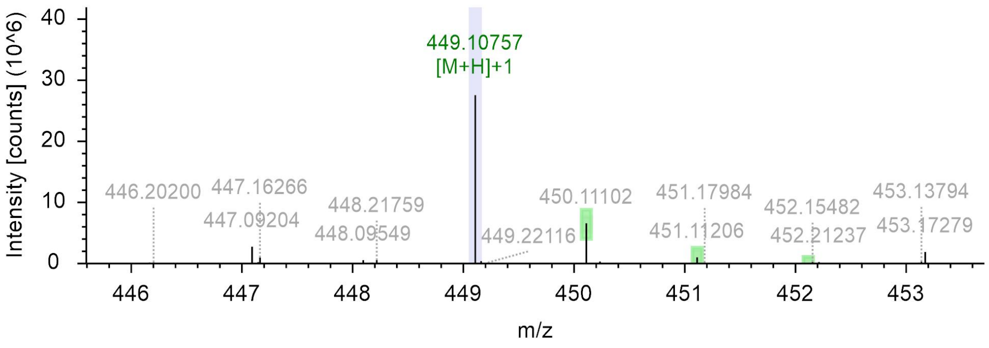

CD111-Sample-20201026 (F952) #2834, RT=6.651 min, MS2, FTMS (-), (HCD, DDF, 447.0938@(20;40;60), -1)

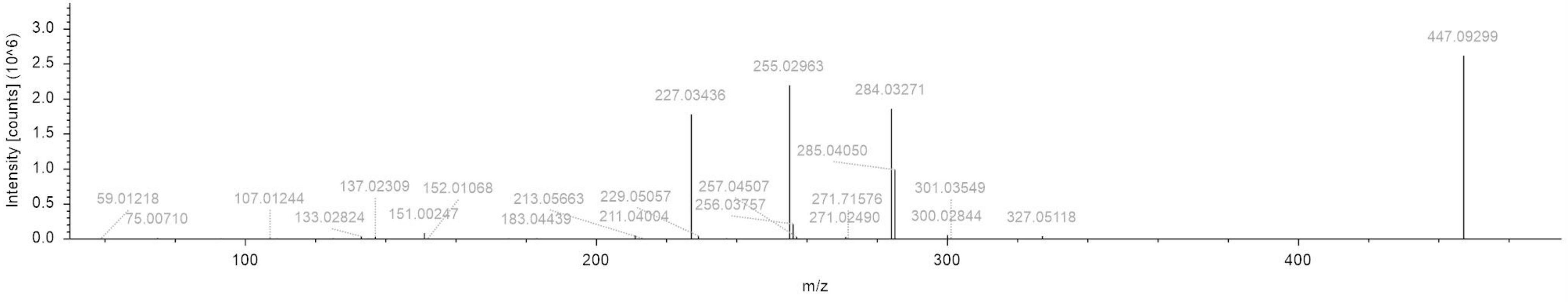

© Reported with Compound Discoverer 2.1

Compounds

03-Nov-2020 11:15

Structure	Name	Formula	FISh Coverage	RT [min]	Molecular Weight	mzCloud Best Match
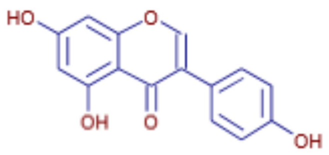	Genistein	C15 H10 O5		8.11	270.0529	88.4

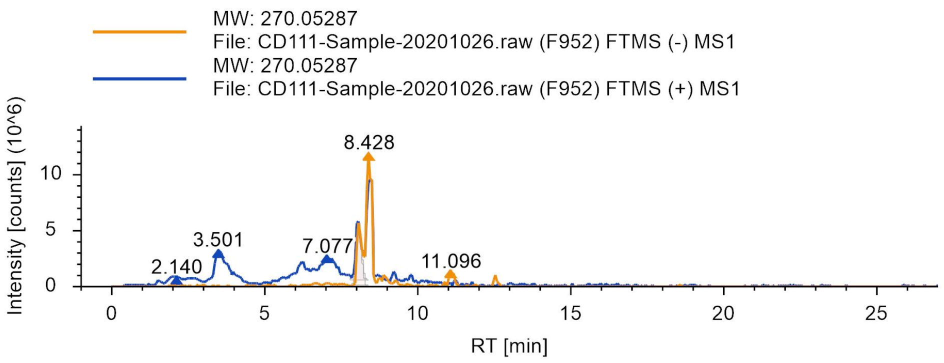

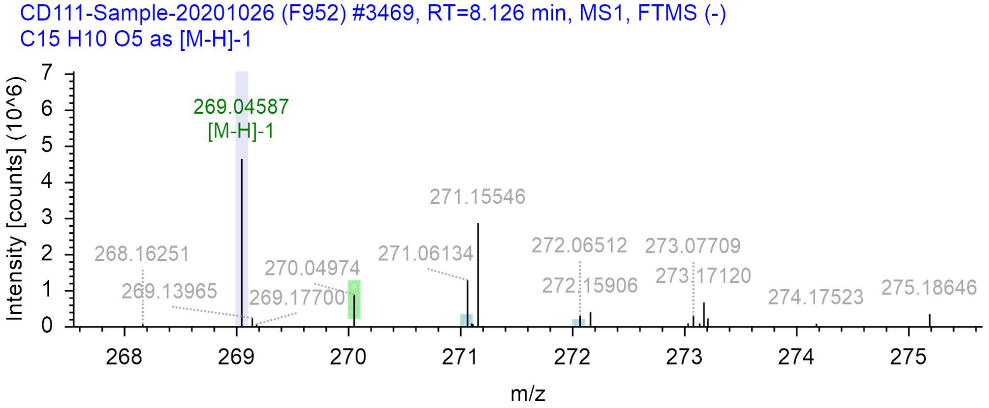

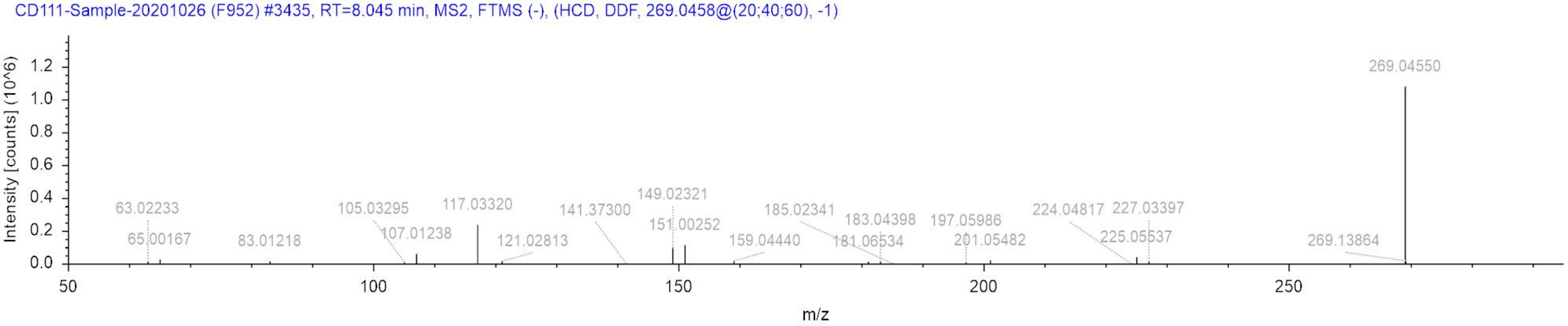

© Reported with Compound Discoverer 2.1

Compounds

03-Nov-2020 11:15

Structure	Name	Formula	FISh Coverage	RT [min]	Molecular Weight	mzCloud Best Match
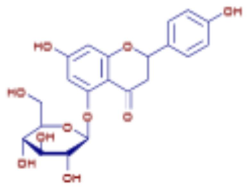	7-Hydroxy-2-(4-hydroxyphenyl)-4-oxo-3,4-dihydro-2H-chromen-5-yl β-D-glucopyranoside	C21 H22 O10		11.29	434.1212	87.9

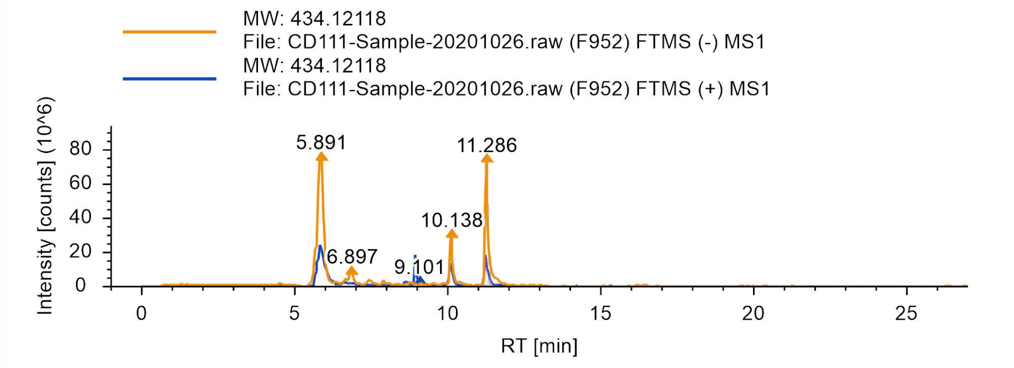

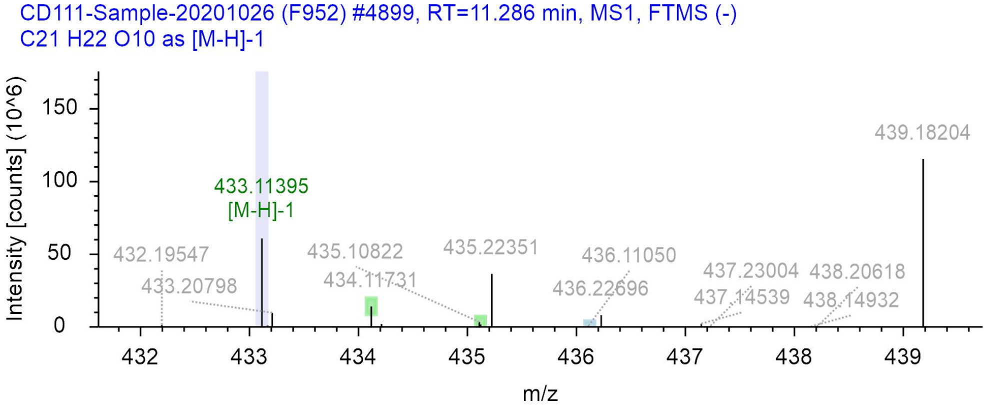

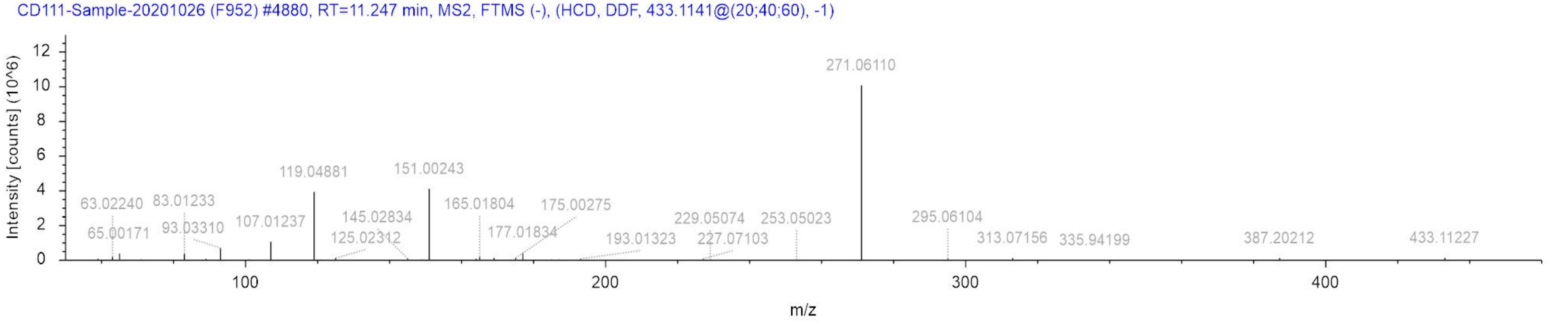

© Reported with Compound Discoverer 2.1

Compounds

03-Nov-2020 11:15

Structure	Name	Formula	FISh Coverage	RT [min]	Molecular Weight	mzCloud Best Match
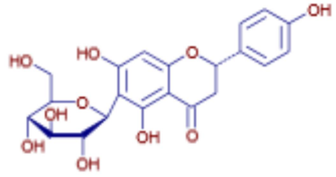	4H-1-Benzopyran-4-one, 6-β-D-glucopyranosyl-2,3-dihydro-5,7-dihydroxy-2-(4-hydroxyphenyl)-, (S)-	C21 H22 O10		5.86	434.1212	86.4

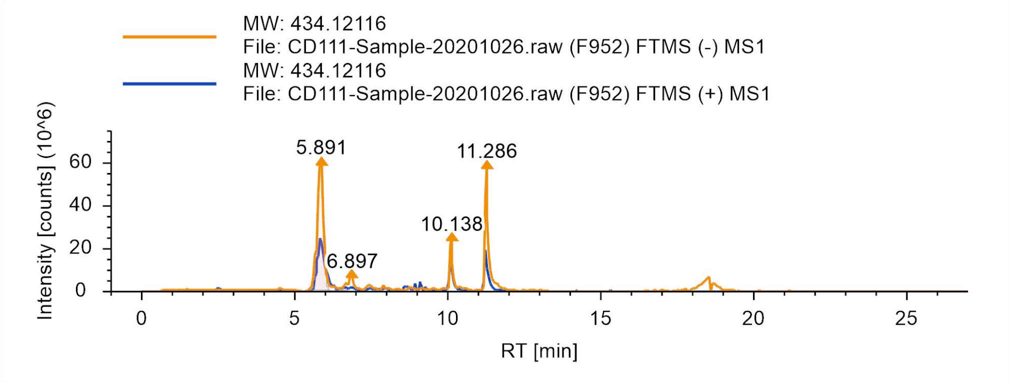

CD111-Sample-20201026 (F952) #2479, RT=5.840 min, MS1, FTMS (-)
C21 H22 O10 as [M-H]-1

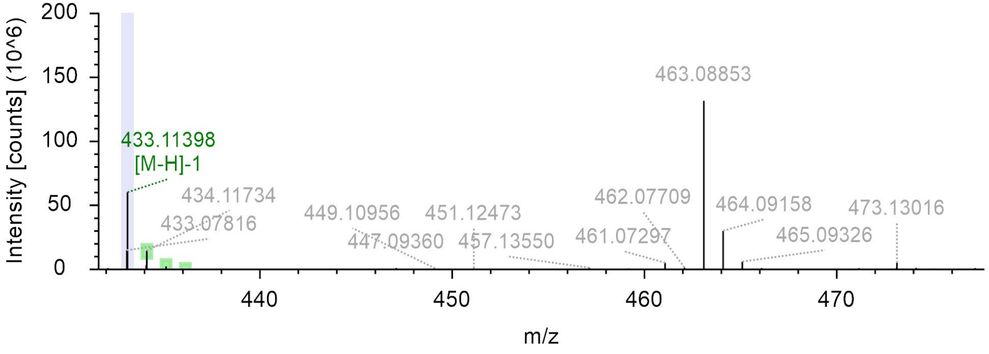

CD111-Sample-20201026 (F952) #2480, RT=5.845 min, MS2, FTMS (-), (HCD, DDF, 433.1139@(20;40;60), -1)

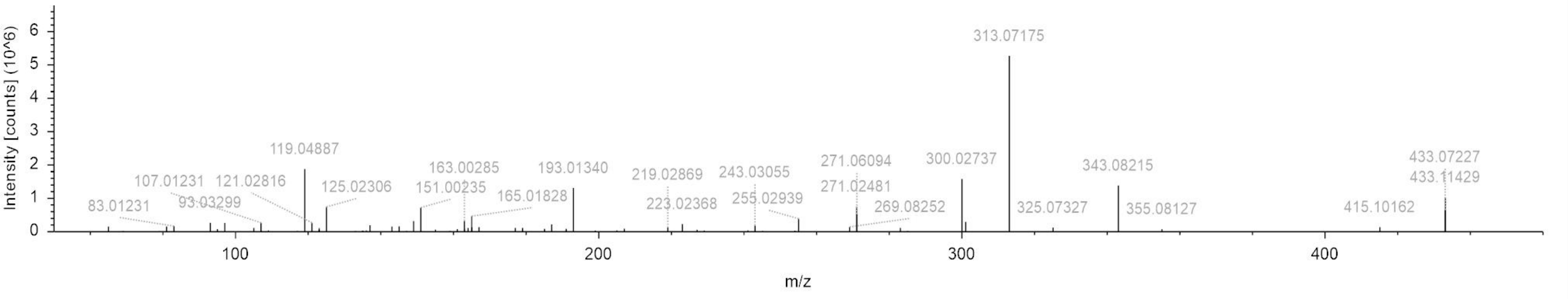

© Reported with Compound Discoverer 2.1

Compounds

03-Nov-2020 11:15

Structure	Name	Formula	FISh Coverage	RT [min]	Molecular Weight	mzCloud Best Match
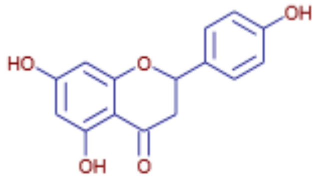	Naringenin	C15 H12 O5		7.74	272.0688	86.3

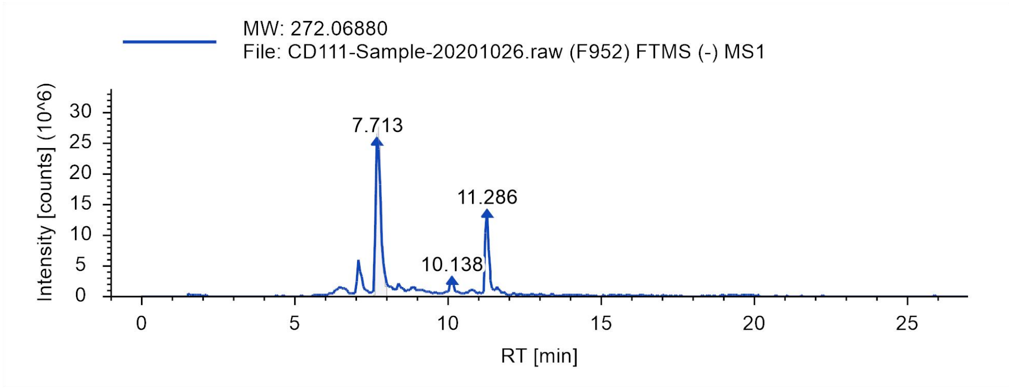

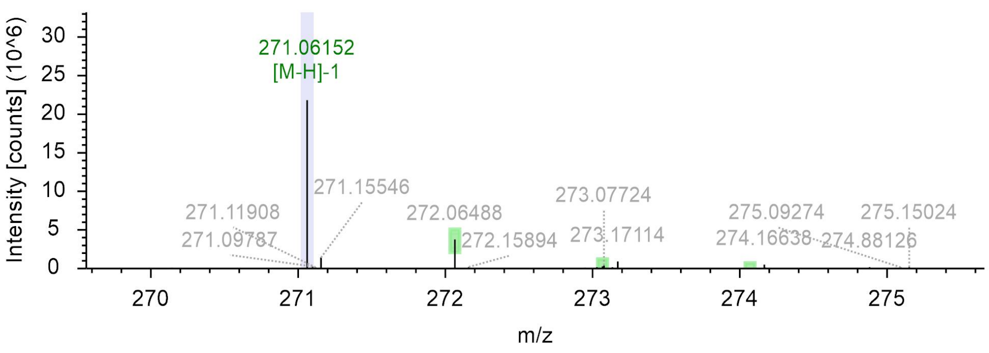

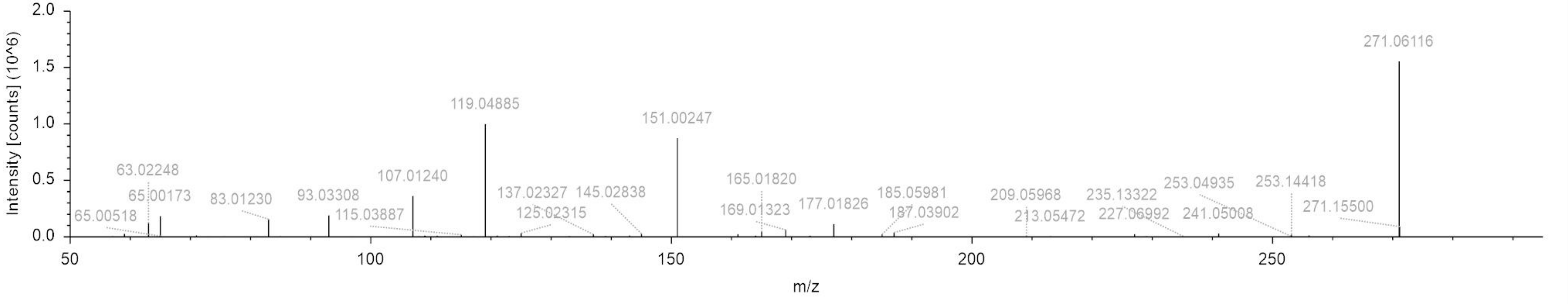

© Reported with Compound Discoverer 2.1

Compounds

03-Nov-2020 11:15

Structure	Name	Formula	FISh Coverage	RT [min]	Molecular Weight	mzCloud Best Match
	Kaempferol-7-O-glucoside	C21 H20 O11		6.20	448.1003	85.5

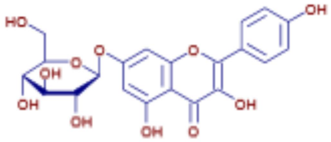

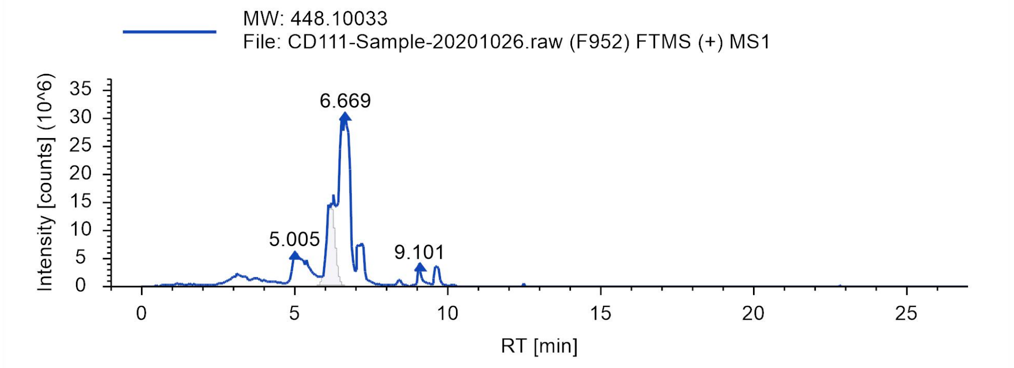

CD111-Sample-20201026 (F952) #2644, RT=6.212 min, MS1, FTMS (+)
C21 H20 O11 as [M+H]+1

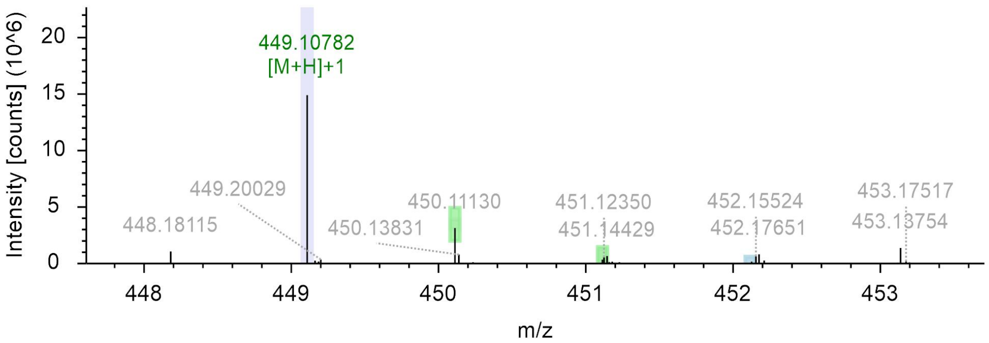

CD111-Sample-20201026 (F952) #2648, RT=6.221 min, MS2, FTMS (+), (HCD, DDF, 449.1078@(20;40;60), +1)

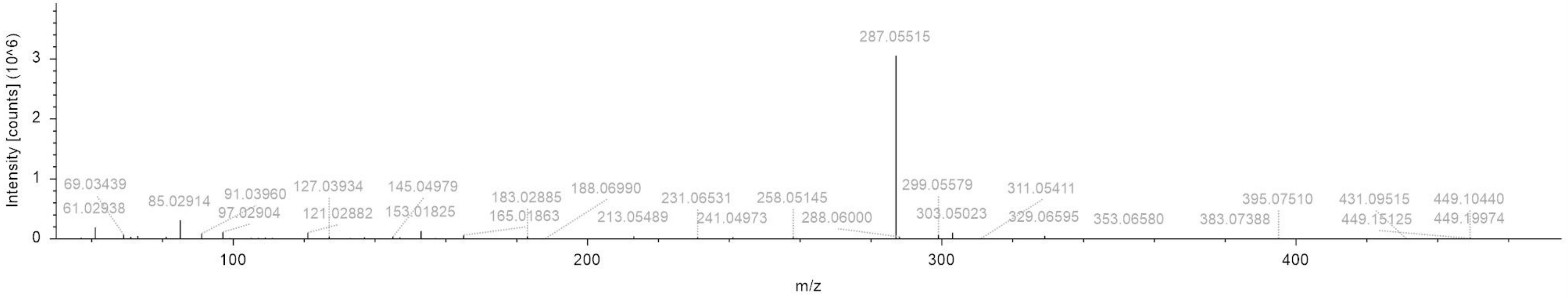

© Reported with Compound Discoverer 2.1

Compounds

03-Nov-2020 11:15

Structure	Name	Formula	FISh Coverage	RT [min]	Molecular Weight	mzCloud Best Match
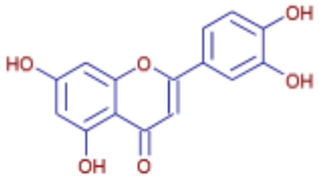	Luteolin	C15 H10 O6		6.63	286.0478	85.3

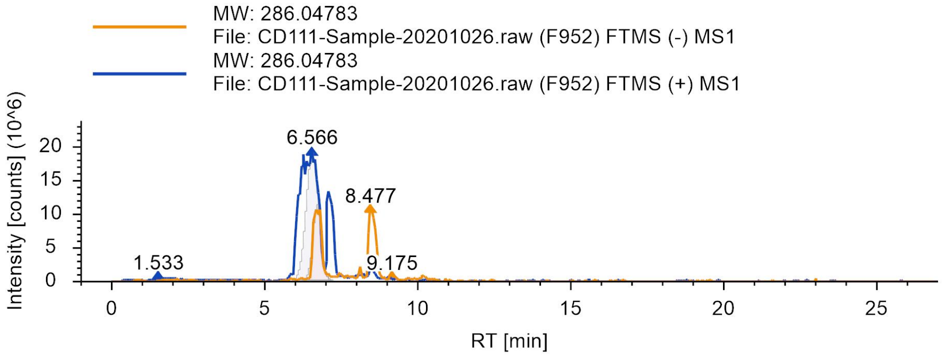

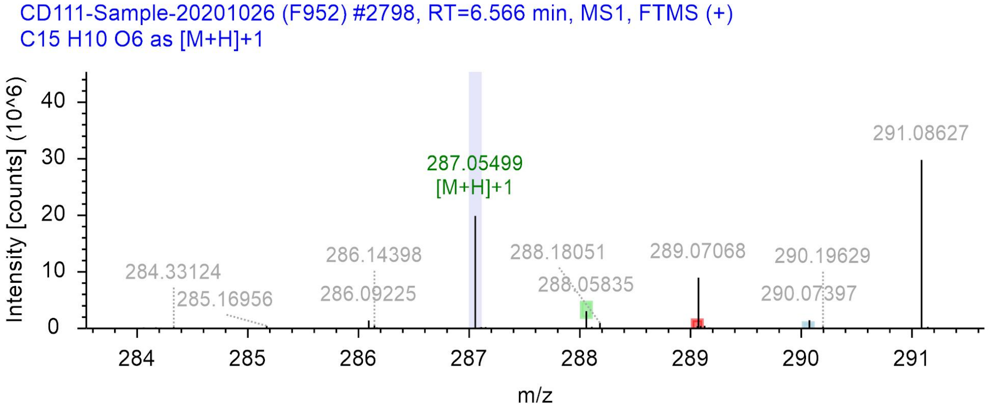

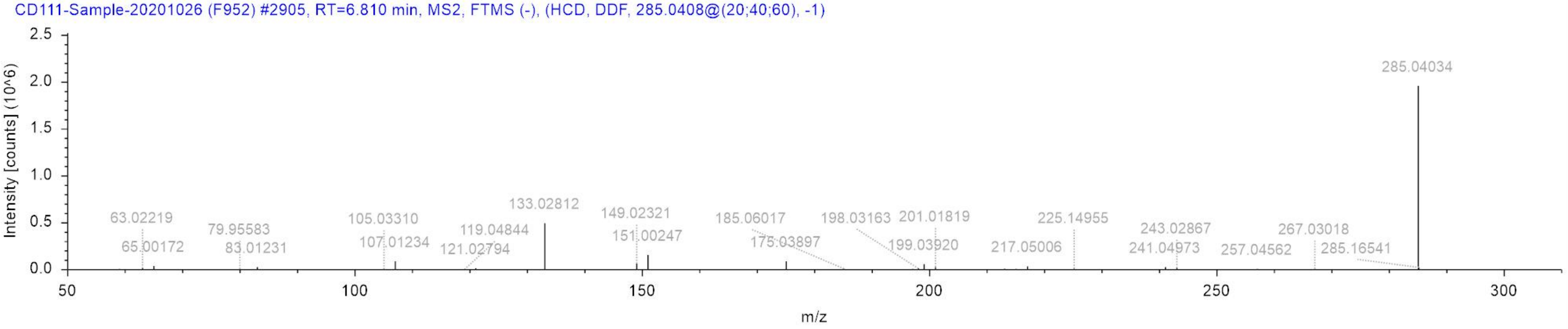

© Reported with Compound Discoverer 2.1

Compounds

03-Nov-2020 11:15

Structure	Name	Formula	FISh Coverage	RT [min]	Molecular Weight	mzCloud Best Match
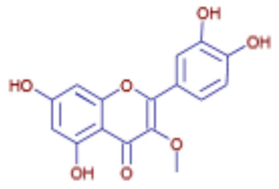	3-Methoxy-5,7,3',4'-tetrahydroxy-flavone	C16 H12 O7		9.07	316.0585	84.0

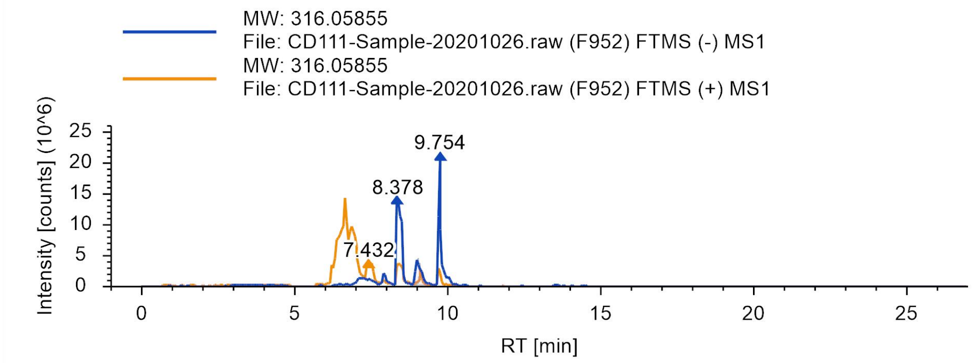

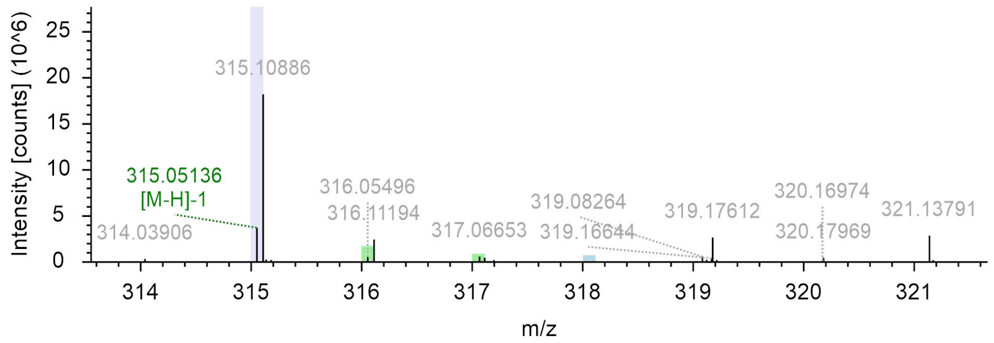

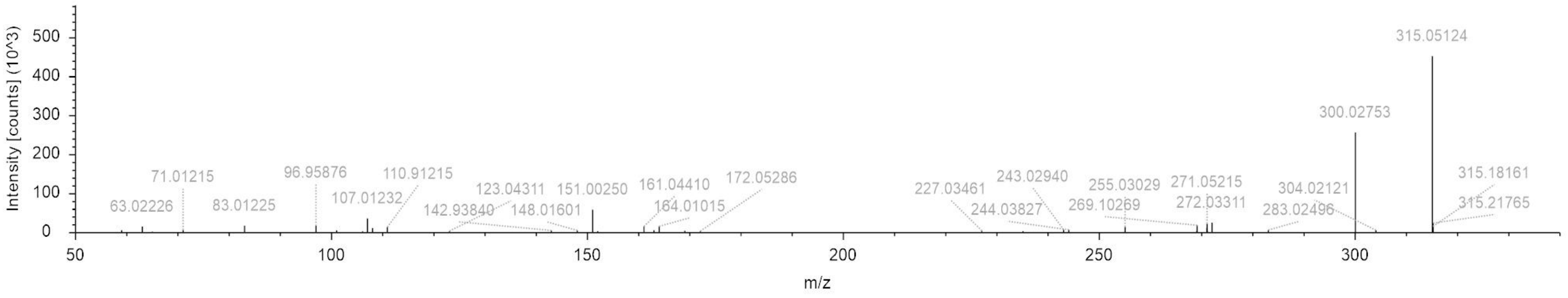

© Reported with Compound Discoverer 2.1

Compounds

03-Nov-2020 11:15

Structure	Name	Formula	FISh Coverage	RT [min]	Molecular Weight	mzCloud Best Match
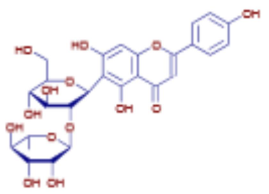	(1S)-1,5-Anhydro-2-O-(6-deoxy-α-L-mannopyranosyl)-1-[5,7-dihydroxy-2-(4-hydroxyphenyl)-4-oxo-4H-chromen-6-	C27 H30 O14		5.14	578.1631	83.7

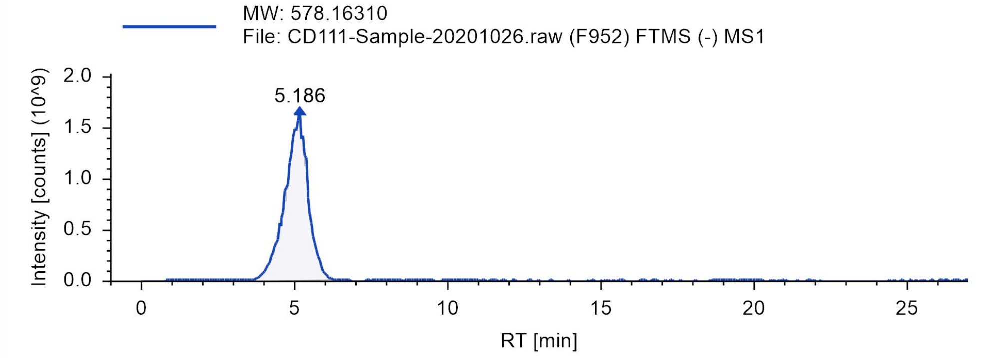

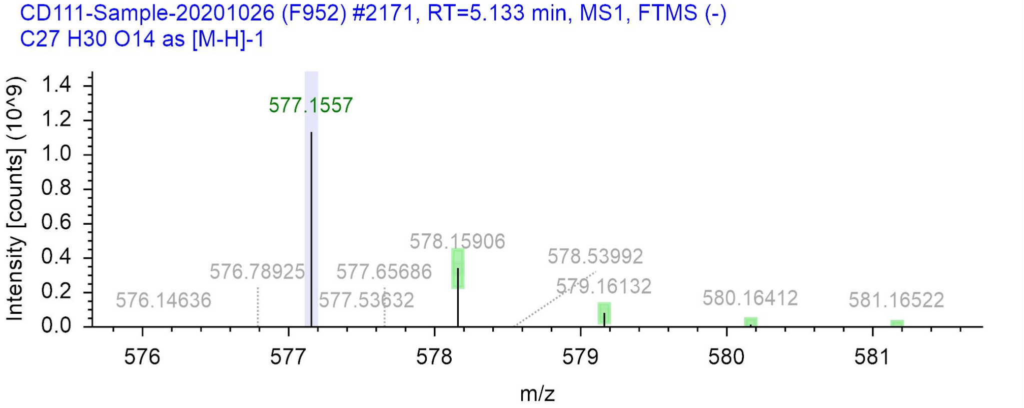

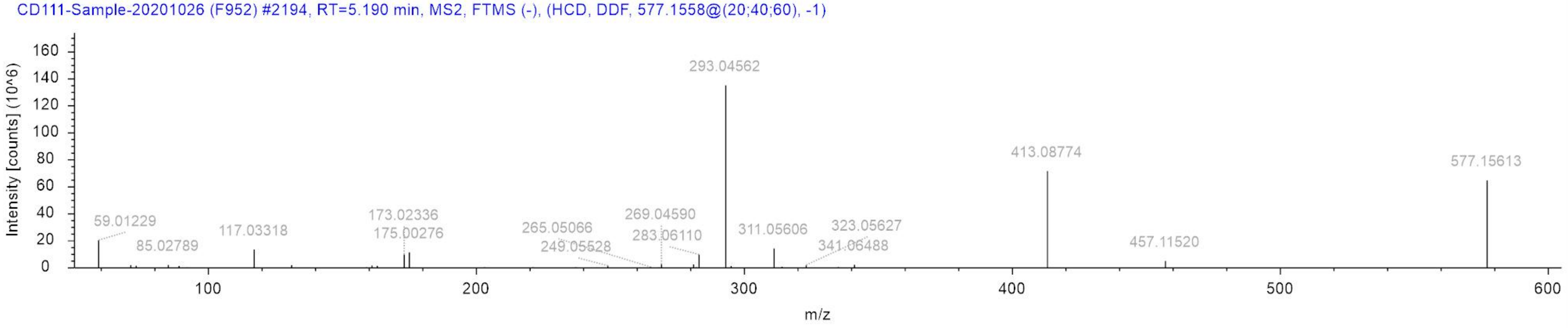

© Reported with Compound Discoverer 2.1

Compounds

03-Nov-2020 11:15

Structure	Name	Formula	FISh Coverage	RT [min]	Molecular Weight	mzCloud Best Match
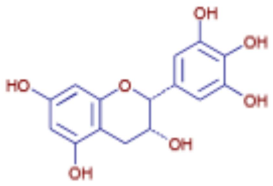	(-)-Epigallocatechin	C15 H14 O7		7.10	288.0634	81.7

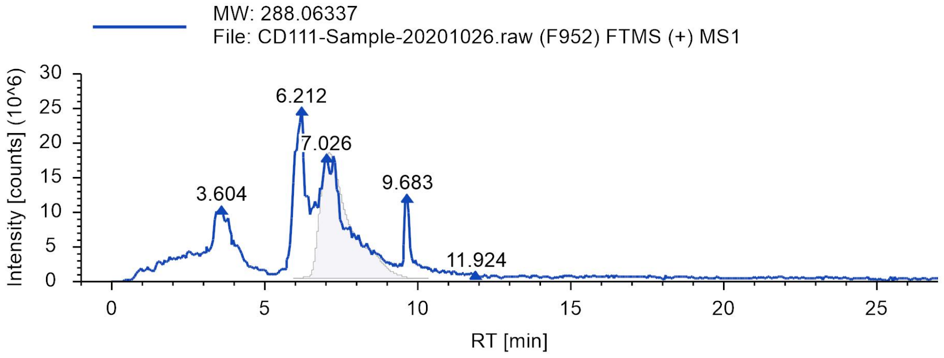

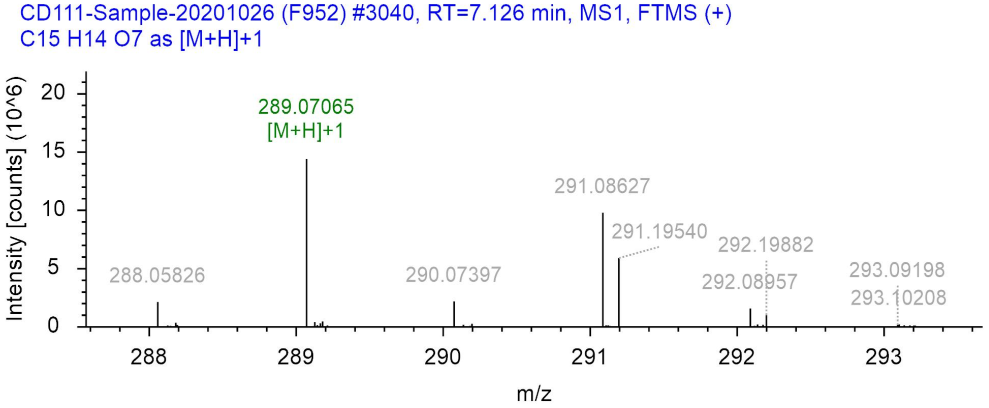

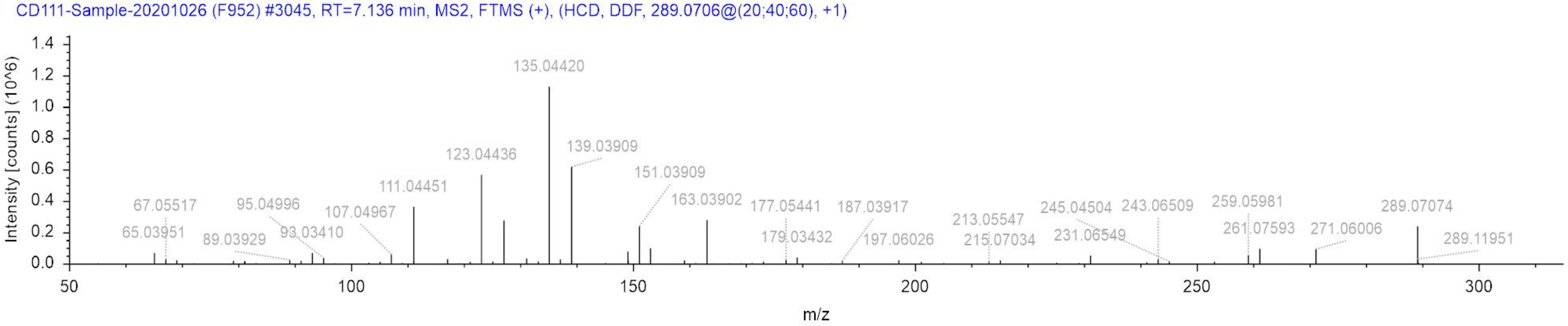

© Reported with Compound Discoverer 2.1

Compounds

03-Nov-2020 11:15

Structure	Name	Formula	FISh Coverage	RT [min]	Molecular Weight	mzCloud Best Match
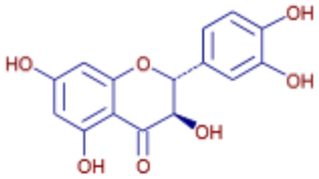	Taxifolin	C15 H12 O7		2.63	304.0557	81.1

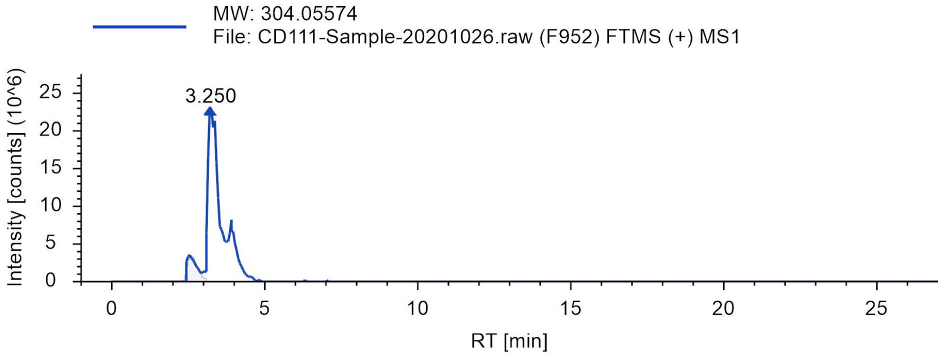

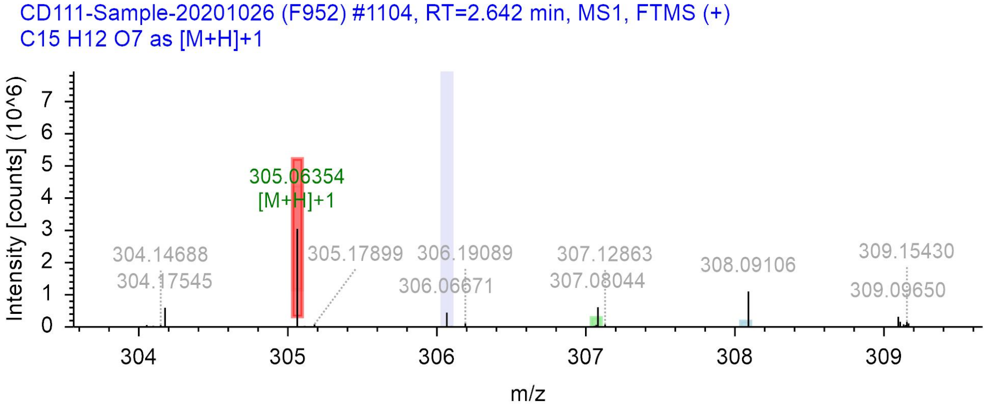

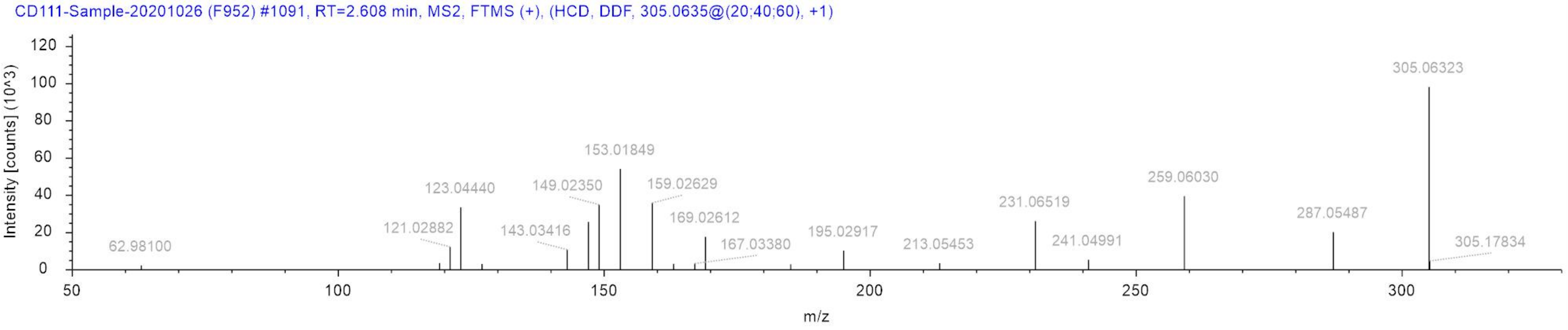

© Reported with Compound Discoverer 2.1

Compounds

03-Nov-2020 11:15

Structure	Name	Formula	FISh Coverage	RT [min]	Molecular Weight	mzCloud Best Match
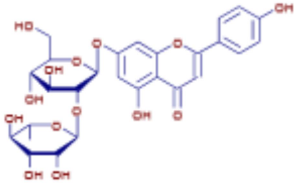	Rhoifolin	C27 H30 O14		5.05	600.1446	81.1

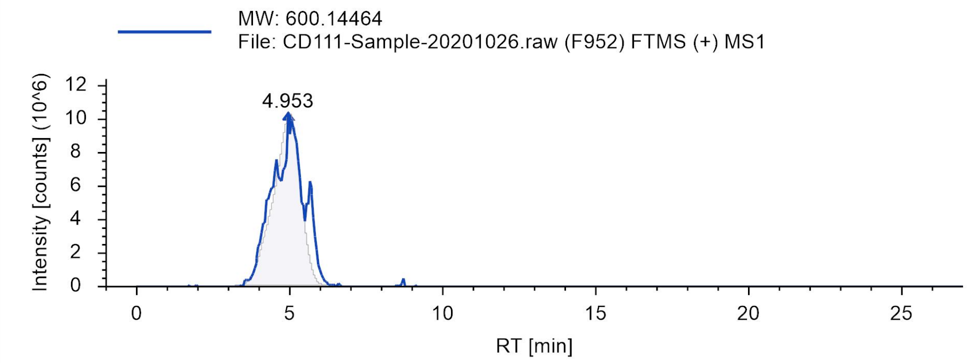

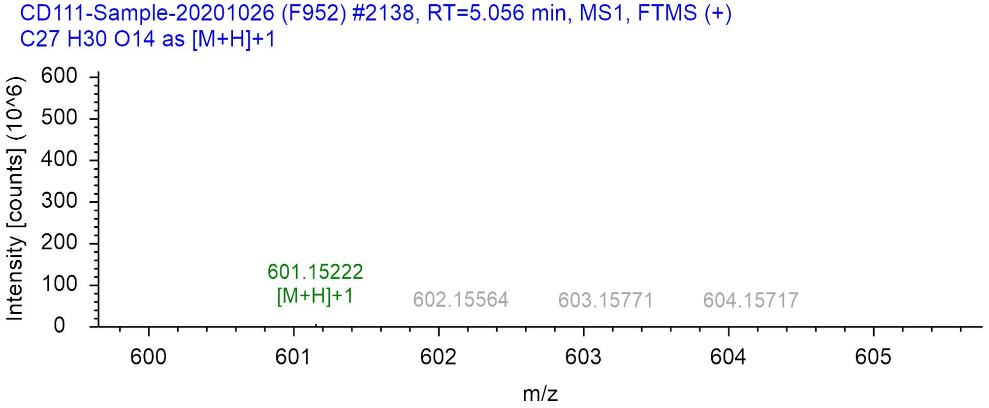

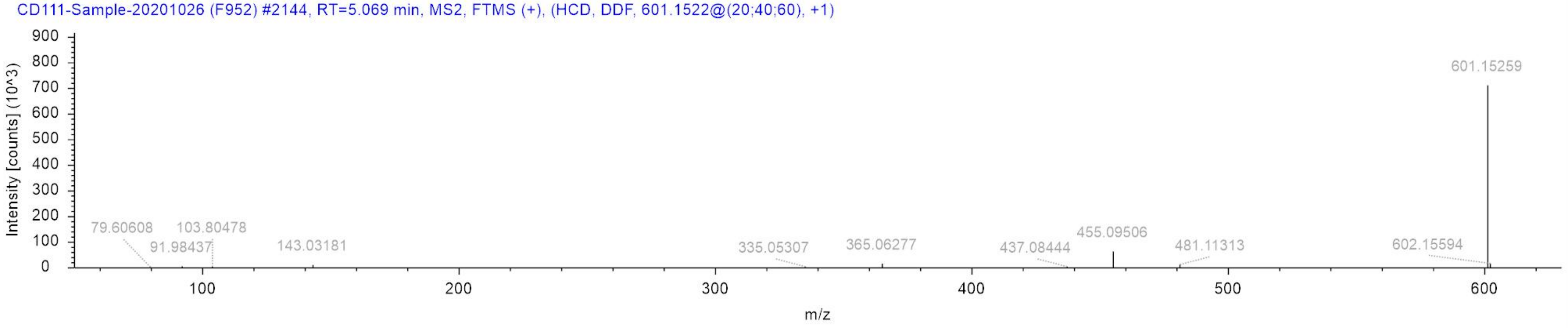

© Reported with Compound Discoverer 2.1

Compounds

03-Nov-2020 11:15

Structure	Name	Formula	FISh Coverage	RT [min]	Molecular Weight	mzCloud Best Match
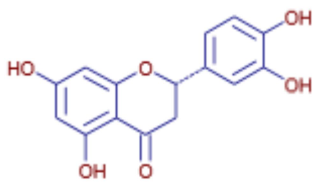	Eriodictyol	C15 H12 O6		9.67	288.0635	80.5

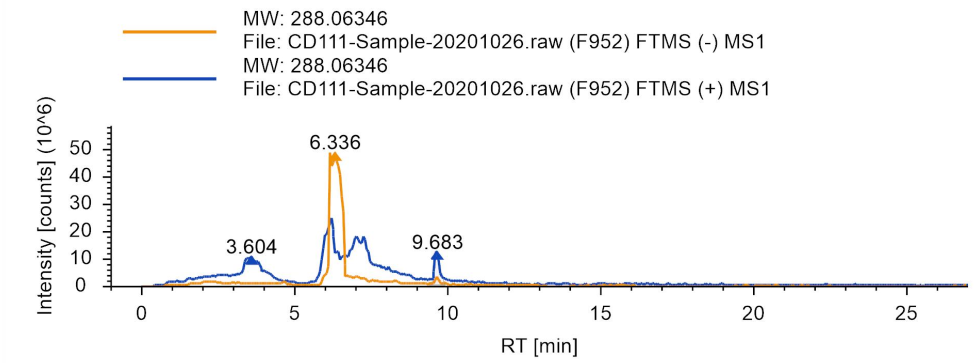

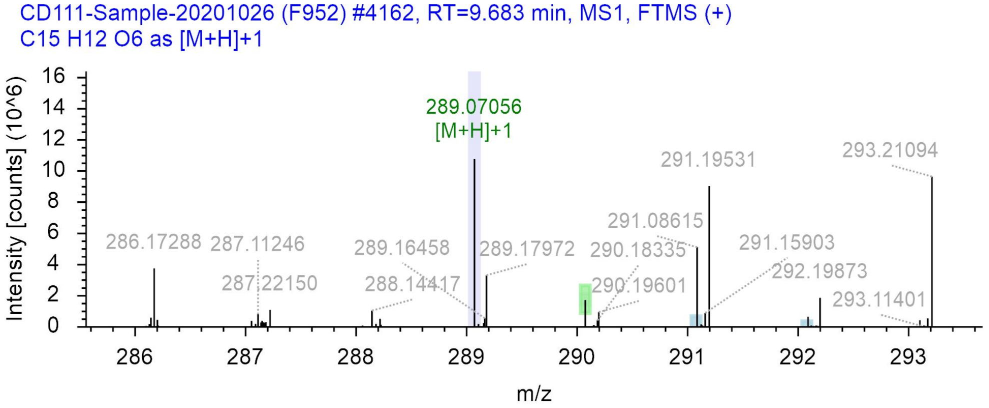

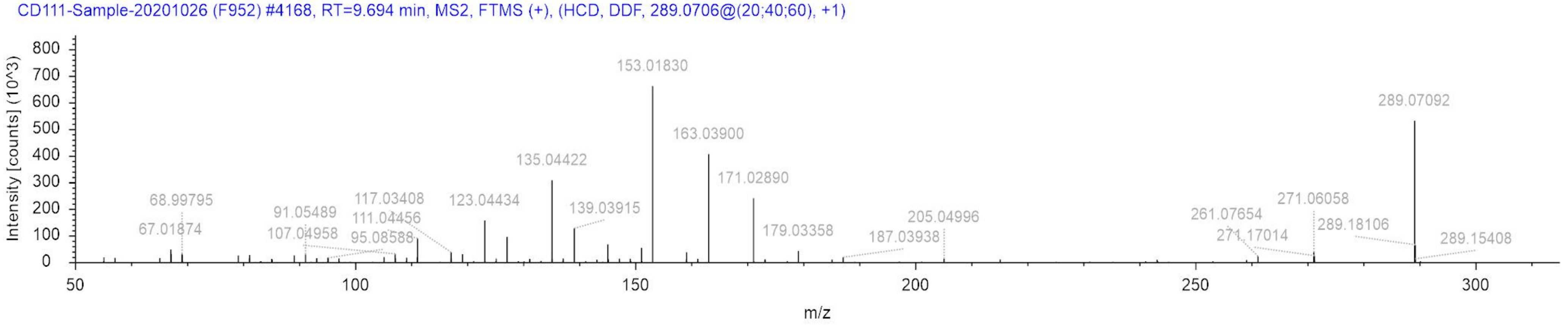

© Reported with Compound Discoverer 2.1
